# Polysaccharide-Based Nanocomposites for Food Packaging Applications

**DOI:** 10.3390/ma14195549

**Published:** 2021-09-24

**Authors:** Kunal Pal, Preetam Sarkar, Arfat Anis, Karolina Wiszumirska, Maciej Jarzębski

**Affiliations:** 1 Department of Biotechnology and Medical Engineering, National Institute of Technology Rourkela, Rourkela 769008, India; 2 Department of Food Process Engineering, National Institute of Technology Rourkela, Rourkela 769008, India; preetamdt@gmail.com; 3 SABIC Polymer Research Center, Department of Chemical Engineering, King Saud University, Riyadh 11421, Saudi Arabia; aarfat@ksu.edu.sa; 4 Department of Industrial Products and Packaging Quality, Institute of Quality Science, Poznań University of Economics and Business, Al. Niepodległości 10, 61-875 Poznań, Poland; karolina.wiszumirska@ue.poznan.pl; 5 Department of Physics and Biophysics, Faculty of Food Science and Nutrition, Poznań University of Life Sciences, Wojska Polskiego 38/42, 60-637 Poznań, Poland

**Keywords:** starch, chitosan, cellulose, nanoparticles, silver nanoparticles, nanocomposites

## Abstract

The article presents a review of the literature on the use of polysaccharide bionanocomposites in the context of their potential use as food packaging materials. Composites of this type consist of at least two phases, of which the outer phase is a polysaccharide, and the inner phase (dispersed phase) is an enhancing agent with a particle size of 1–100 nm in at least one dimension. The literature review was carried out using data from the Web of Science database using VosViewer, free software for scientometric analysis. Source analysis concluded that polysaccharides such as chitosan, cellulose, and starch are widely used in food packaging applications, as are reinforcing agents such as silver nanoparticles and cellulose nanostructures (e.g., cellulose nanocrystals and nanocellulose). The addition of reinforcing agents improves the thermal and mechanical stability of the polysaccharide films and nanocomposites. Here we highlighted the nanocomposites containing silver nanoparticles, which exhibited antimicrobial properties. Finally, it can be concluded that polysaccharide-based nanocomposites have sufficient properties to be tested as food packaging materials in a wide spectrum of applications.

## 1. Introduction

In the packaging industry, synthetic films are used, which are made of petroleum products. Properly designed packaging materials should enable the mechanical recycling process. Unfortunately, there are many barriers, such as multi-material packaging materials and contamination with product residues. This kind of waste cannot be recycled and processed into another packaging material or industrial product, e.g., fibers, garden furniture, building materials, etc. This ultimately results in the loss of valuable raw material and the non-closing of the loop [1]. The disposal of these kinds of packaging materials is a serious environmental problem, and irresponsible management increases the environmental footprint. An opportunity for the packaging industry is biodegradable materials that meet detailed standard requirements. It is important to note that biodegradable materials are already being used in many food industries to develop various items that include, but are not limited to, straws, cutlery, cups, and containers [2].

**Figure 1:**
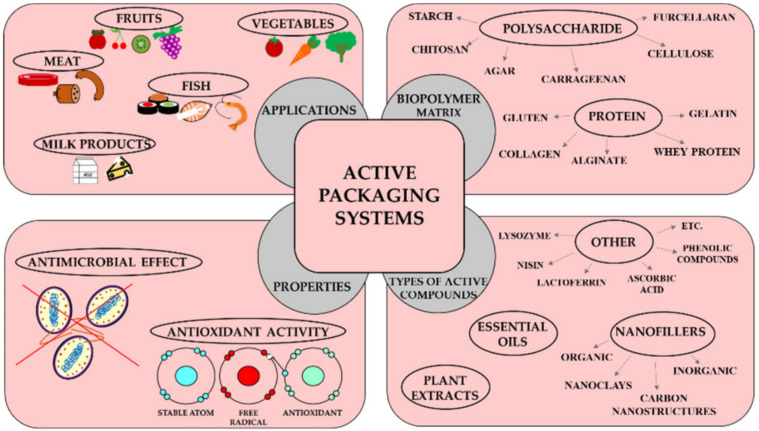
The active properties of biopolymer films as the main compounds in active packaging materials. (Reproduced under Creative Commons License from [7]).

The materials that are being obtained from nature are mostly biodegradable. The polymers that are obtained from nature are regarded as biopolymers. They are usually classified as polysaccharides (e.g., chitosan, alginate, starch, and agar) and proteins (e.g., whey protein isolate, pea protein, and gelatin). A summary of biopolymers films and their properties and applications are provided in Figure 1. In recent years, biopolymers have evolved as an alternative to synthetic films. Some of the commonly explored properties include reducing the water vapor transmission rate, the barrier to the UV and visible radiations, the barrier to the permeation of oxygen, and controlling the loss of the aroma from the food products [3,4,5,6].

Additionally, as the biopolymers are available in nature in abundance and are mostly extracted from the waste or by-products of food or other industries, the cost of the biopolymers is quite cheap. However, many proteins need special and careful handling, which can be quite tricky while developing the packaging materials. On the other hand, most of the polysaccharides (e.g., chitosan, alginate, starch, cellulose, and pectin) can be easily processed to develop films [7,8].

The film-forming ability is an essential criterion for developing food packaging materials and food coatings. However, the films of polysaccharides are quite brittle and exhibit poor water resistance and mechanical properties. The brittle nature of the polysaccharide-based films can be overcome by adding plasticizers (e.g., glycerol, polyethylene glycol, fatty acid esters, triglycerides, and phospholipids), which helps make the films ductile. Various oligosaccharides like fructose-glucose syrups, sucrose, glucose, and honey have also been explored as plasticizers by many researchers [9]. They also reduce the chances of deformation of the films during the preparation stage. The addition of plasticizers has also been related to the water vapor permeation capability of the films. The hydrophilic plasticizers increase water vapor permeation, while the hydrophobic plasticizers have been reported to reduce the permeation rate [10,11] significantly.

The mechanical properties of the polysaccharide films can be improved by reinforcing materials. The reinforcing materials remain as a distinct and dispersed phase when incorporated within the polysaccharide phase. The reinforcement can be carried out in three ways, particle reinforcement, short fiber reinforcement, and continuous fiber reinforcement [12]. The nanocomposites extend better mechanical and physical properties over the polymeric films [13,14]. The enhanced properties of the nanocomposites have been explained by the very high surface-to-volume ratio [15]. Polysaccharide-based nanocomposites (PNCs) have been explored as biodegradable packaging materials for food products. Many nanomaterials (e.g., titanium oxide nanoparticles, zinc oxide nanoparticles, silver nanoparticles, gold nanoparticles, and copper oxide nanoparticles), which are used as the reinforcement material, can also impart antimicrobial properties to the PNCs [16,17,18,19]. These properties of PNCs make them suitable for food packaging applications. A large number of researchers have employed polysaccharides, namely chitosan, pectin, starch, and cellulose, to design food packaging materials based on PNCs.

In the present review, a scientometric analysis is initially carried out using the Web of Science (WoS) database. The scientometric data is evaluated to understand the evolution of the research across the globe. The widely studied polysaccharides and nanomaterials for food packaging applications are then subsequently identified. The properties, synthesis of PNCs, and applications of these materials are then discussed in detail. It should also be noted that all materials intended for use in direct contact with food must meet a number of legal requirements in force in various regions of the world, e.g., Europe, the USA, Mercosur, and China. They also require certification confirming biodegradability and compostability, among others: ASTM D6400, ASTM D6868, EN 13432, EN 14995, EN 17033, ISO 17088, ISO 18606, NF T 51-800, AS 4736, AS 5810.

## 2. Scientometric Analysis

The WoS Core Collection database was accessed on June 13, 2021. The Science Citation Index-Expanded index was only searched. No restrictions on the timespan were employed. The database was searched with the keywords: TOPIC: (“nanocomposite*”) AND TOPIC: (“food packaging”). The search returned with 1406 publications. The publications were categorized as article (1200 publications), early access (40 publications), book chapter (2 publications), meeting abstract (6 publications), review (198 publications), proceedings paper (32 publications), and editorial material (2 publications). In our study, only articles thathad 1199 publications were selected for further analysis. The publications were written in the language of English (1194 publications), Portuguese (4 publications), Korean (1 publication), and Spanish (1 publication). The publications in the English language were only selected for further analysis. Hence, finally, 1194 publications were returned for scientometric analysis.

**Figure 2:**
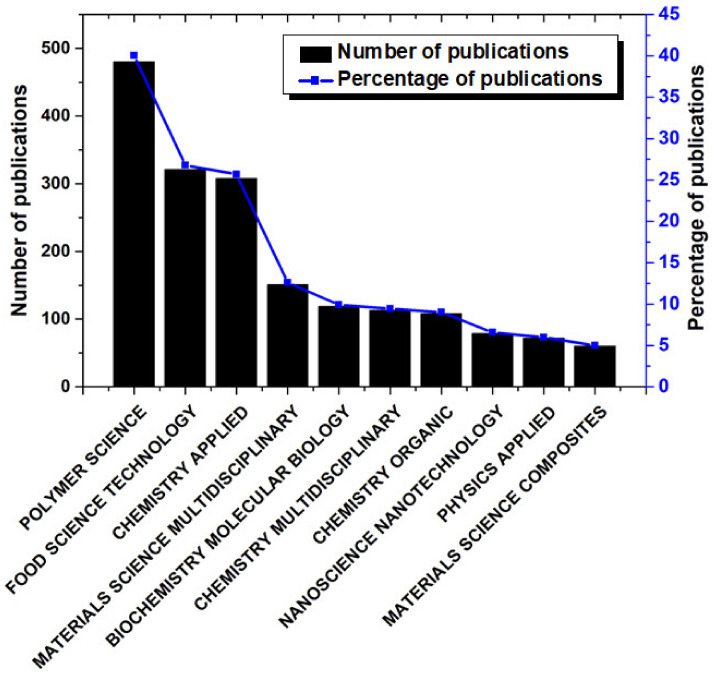
Distribution of the research articles based on WoS categories.

**Figure 3:**
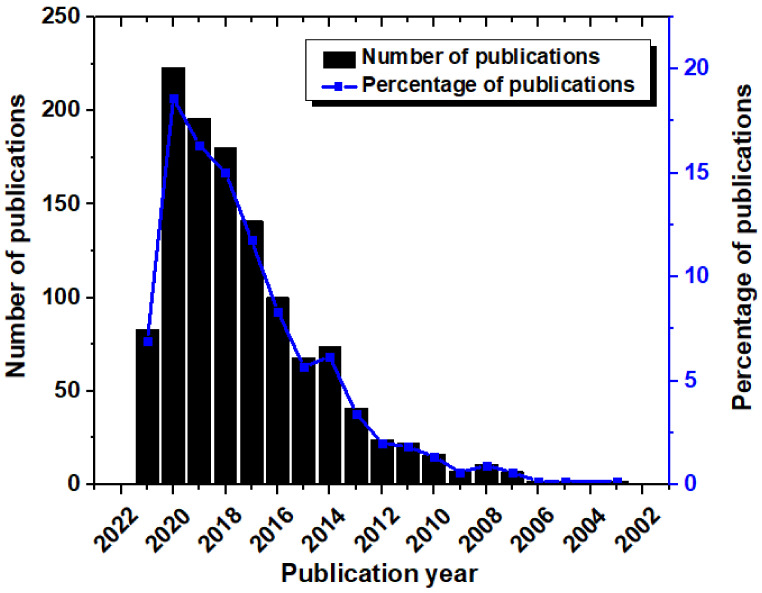
Yearwise distribution of the publications.

At the initial stage, the preliminary analysis was carried out by the “Analyze Results” tool of the WoS. The analysis of the WoS categories suggested that the publications on the nanocomposite-based food packaging materials were mainly published in three main categories. These categories include polymer science, food science and technology, and applied chemistry (Figure 2). The highest number of publications was published in the field of polymer science, where 480 publications were published. This constituted 40.03% of the total publications. Thereafter, the two most significant WoS categories were food science and technology and applied chemistry, where 321 (26.77%) and 308 (25.69%) publications were published, respectively. In the field of materials science, 151 publications were published, which constituted 12.59% of the total number of publications. The percentage of publications in the rest of the categories was lower than 10%. The analysis of the publications over the years provides an indication of the growth of the research field. Hence, the number of publications with respect to the publication year was plotted (Figure 3). It could be observed that during the year 2003, two publications based on nanocomposite food packaging materials were first published. From then till the year 2010, the growth in the number of publications was not significant. Post year 2010, there has been an exponential increase in the number of publications related to nanocomposite food packaging materials. This can be explained by the increase in the concerns and consequent steps taken by the respective governments to improve the status of food security and food safety in their countries. It can also be observed from Figure 3 that there is a corresponding increase in the percentage of publications in the said field of research as the years are progressing.

**Figure 4:**
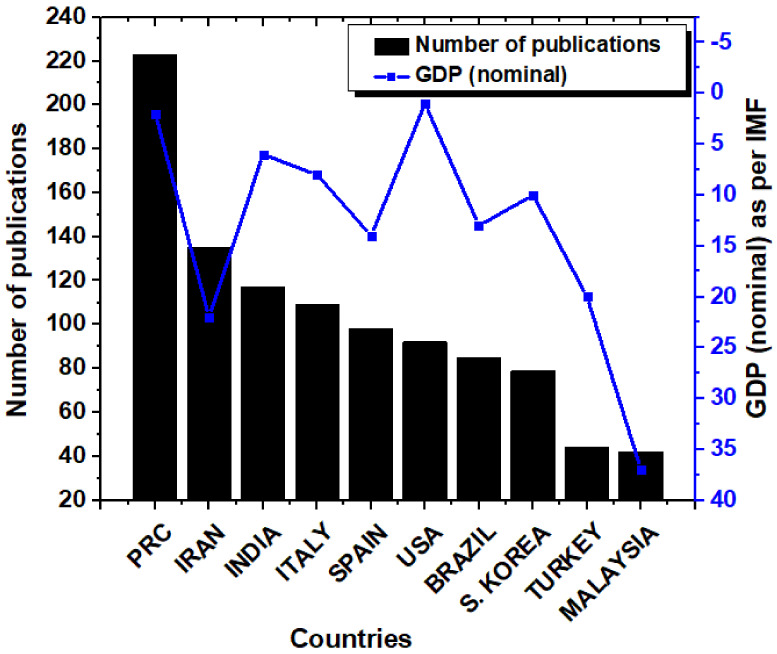
Countrywise distribution of the publications and the GDP (nominal) of the countries.

Figure 4 summarizes the top 10 countries with the maximum number of publications and the gross domestic product (GDP) (Nominal) of the countries as per the International Monetary Fund (IMF). Countrywise distribution of the publications is an indicator of the funding being invested by a particular government in a particular research field. The countries with the major numbers of publications were The People’s Republic of China (PRC) (223 publications), Iran (135 publications), India (117 publications), Italy (109 publications), and Spain (98 publications). It can be seen that the number of publications from the PRC supersedes the number of publications from any other country. The United States of America (USA) was at a distant sixth position with only 92 publications. Except for Iran, Turkey, and Malaysia, all the other countries among the top 10 countries had a GDP (Nominal) of less than 15. This is suggestive of the fact that most of the economically superior countries were investing money in the research for nanocomposite-based food packaging systems. It also indicates that governments across the globe are serious in overcoming the challenges that are related to food security and food safety.

**Figure 5:**
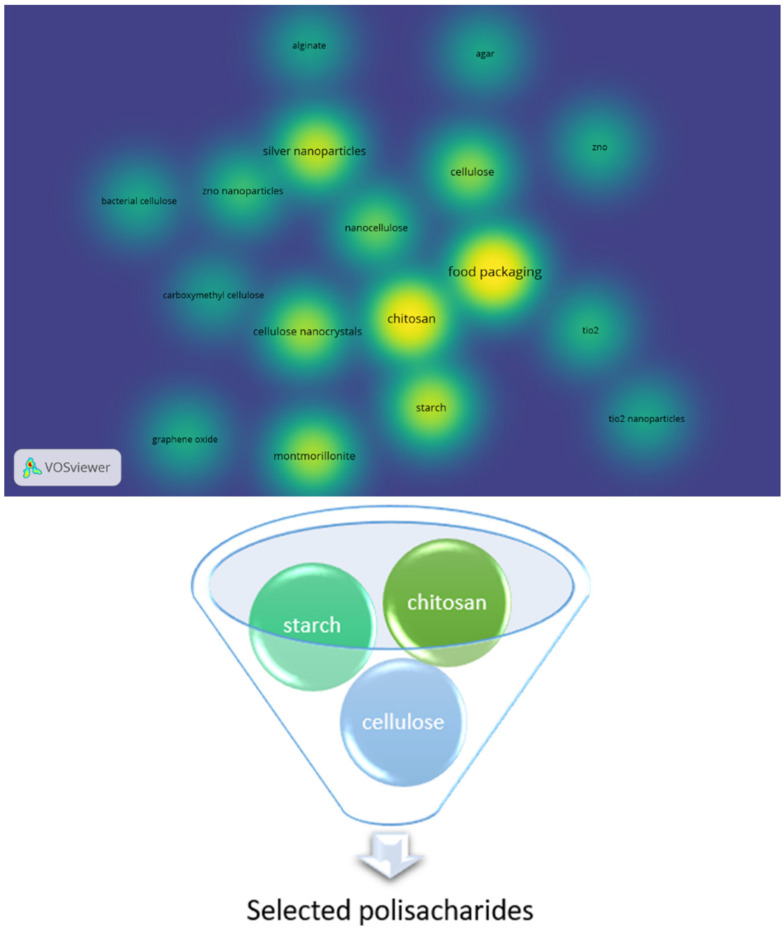
Density plot of the manually selected keywords of polysaccharide and nanoparticles.

The most studied polysaccharides and nanomaterials were identified from the “density plot,” which was created using the VosViewer^®^ free software (Version: 1.6.17 for Microsoft Windows systems; Centre for Science and Technology Studies, Leiden University, Leiden, The Netherlands). For this purpose, the “Full Record” information of the publications was exported and saved as a text file. Since the WoS search engine allows exporting only 500 records at a time, three text files are generated to gather the information of the selected publications. Then, the files were used to generate a density map that is based on the bibliographic data. Cooccurrence analysis was employed on “All keywords” to identify the most studied polysaccharides and nanomaterials. The number of total keywords identified by the software was 3813. The keywords that appeared at least 20 times were kept for further processing. This resulted in the reduction of the keywords to 125. After that, the manual selection of the keywords was carried out. The keywords “food packaging” and names of any polysaccharide and nanomaterial were retained during the manual selection. The other keywords were rejected. The density map of the manually selected keywords is represented in Figure 5. Then, the important polysaccharides and nanomaterials were identified from the density plot. The important polysaccharides were found to be chitosan, starch, and cellulose, while the important nanomaterials were silver nanoparticles, cellulose nanostructures (e.g., cellulose nanocrystals and nanocellulose), and montmorillonite. Accordingly, in this review, the nanocomposites of these identified polysaccharides and nanomaterials will be discussed in detail.

## 3. Chitosan-Based Nanocomposites

**Figure 6:**
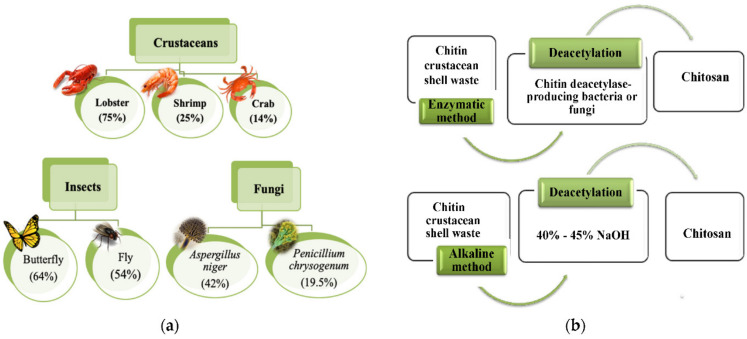
(**a**) Examples of chitin content from different sources, (**b**) Chitin deacetylation methods to produce chitosan. (Reproduced from [36]).

**Figure 7:**
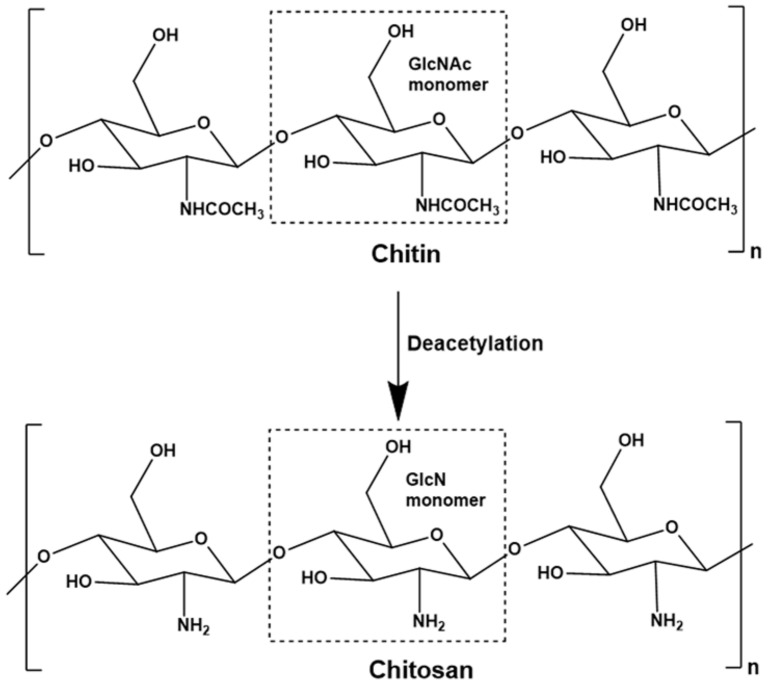
Mechanism of conversion of chitin to chitosan. (Reproduced from [25]).

**Figure 8:**
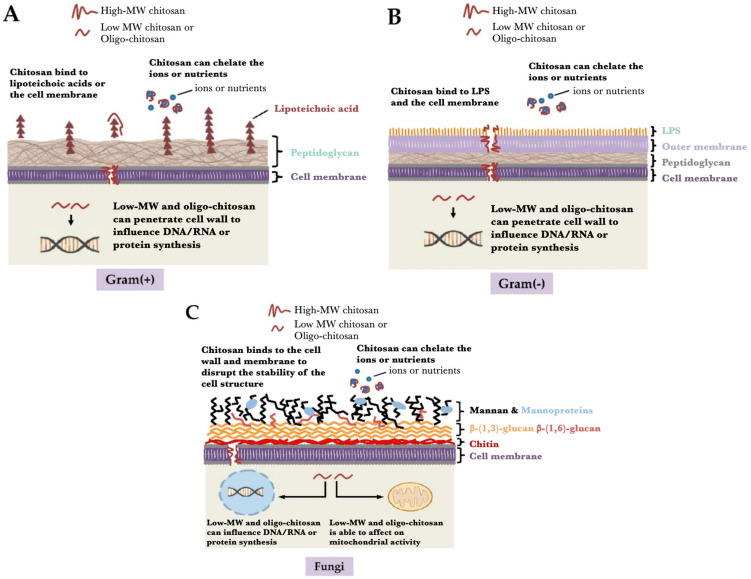
Antimicrobial action of chitosan against (**A**) Gram +ve bacteria, (**B**) Gram -ve bacteria, and (**C**) Fungi. (Reproduced from [37]).

Chitosan has evolved as a polymer that has been exploited in the agriculture, food products, pharmaceutical products, cosmetics, and biomedical industries [20,21,22,23,24]. Chemically, chitosan is represented as a random mixture of β-1,4-linked glucosamine and N-acetylglucosamine. The polymer is categorized as a linear polysaccharide. After cellulose, it is one of the most abundant polysaccharides. The polymer is mainly obtained from crustaceans, shrimps, and fungi (Figure 6a). It is obtained from the polysaccharide named “Chitin,” which is insoluble in water. The process of deacetylation is employed to convert chitin to chitosan. Deacetylation may either be carried out by chemical treatment (alkali treatment) or enzymatic method (Figure 6b). The mechanics of the conversion of chitin to chitosan are represented in Figure 7 [25]. Though the enzymatic treatment method is environment-friendly, the yield of chitosan formed is lower than the chemical treatment method. This polysaccharide is an antimicrobial polymer, which also exhibits antioxidant properties [26,27,28,29]. These properties have been related to the primary amine groups that are abundantly present in chitosan. The presence of the primary amine group allows the polysaccharide to accept a proton at lower pH. The resulting product is regarded as protonated chitosan. Due to this reason, chitosan is categorized as cationic polyelectrolyte. The cationic polymeric chains of chitosan interact with the cell wall of the microbes, which are anionic, and consequently destroy the integrity of the cell wall. This significantly damages the semi-permeable nature of the microbial cell walls and makes them highly permeable, which results in the leakage of the internal (cytosolic) contents from within the microbes. Low molecular chitosan molecules have been reported to penetrate through the cell wall and interact with the genetic material of the microbes, which are also anionic [30]. A summary of the mechanism of the antimicrobial action of chitosan is shown in Figure 8. Also, since the polymer is naturally derived, it is non-toxic and biodegradable. Further, the films of chitosan exhibit barrier properties against water vapor transmission and oxygen permeation [31,32]. The reduction in the permeation of the oxygen allows minimizing the respiration within the fruits and vegetables, which helps to increase the shelf life of the food products [33]. The chitosan-based films and coatings have also been used to improve the shelf life of fish and meat products [34]. However, the properties of the chitosan films and coating can be improved by developing nanocomposite films. Further, improvements in the mechanical properties of the chitosan films have been proposed by developing chitosan/fibroin laminates [35]. This section will discuss the different types of chitosan-based nanocomposites and their applications in food preservation.

### 3.1. Chitosan and Silver Nanoparticle-Containing Food Packaging Systems

**Figure 9:**
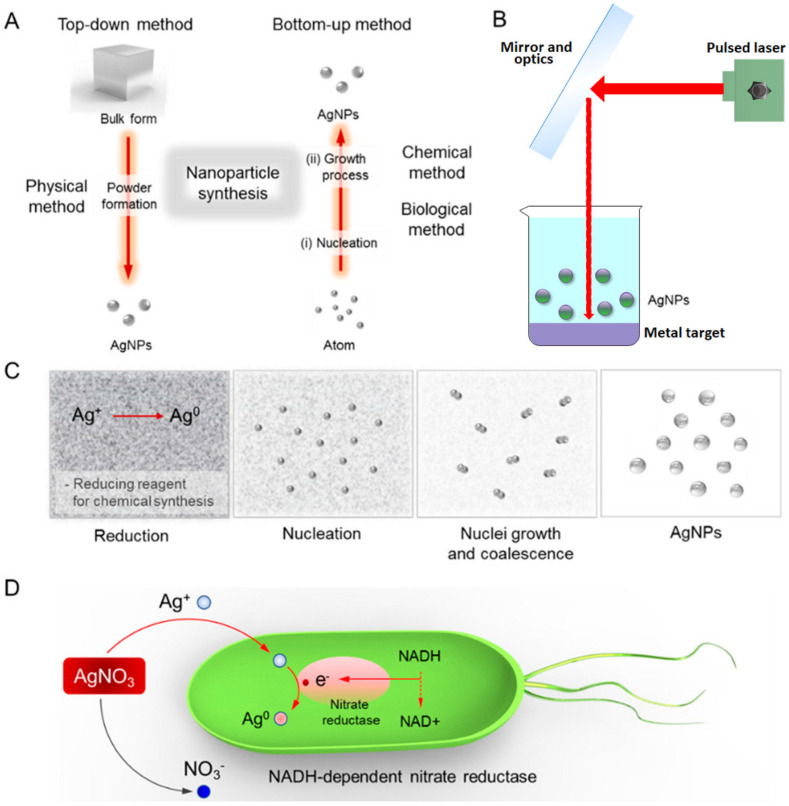
Diverse synthesis routes of silver nanoparticles (AgNPs). (**A**) Top-down and bottom-up methods. (**B**) Physical synthesis method. (**C**) Chemical synthesis method. (**D**) Plausible synthesis mechanisms of green chemistry. (Reproduced from [42]).

**Figure 10:**
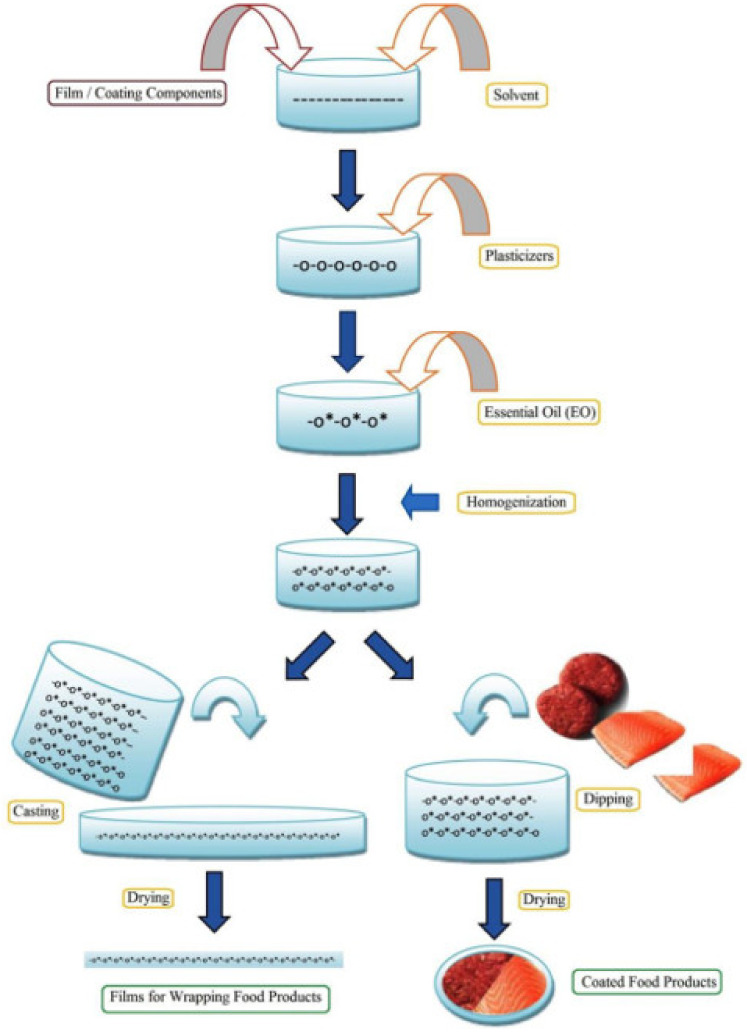
Schematic representation of the solution casting method. (Reproduced from [43]).

The inclusion of silver nanoparticles within polymer matrices has been reported to impart broad-spectrum antimicrobial property to the nanocomposites [38]. The different modalities of silver nanoparticle systems are summarized in Figure 9. The nanoparticles have been reported to prevent the adhesion of the microbes onto the substrate. The antimicrobial activity of the silver nanoparticles has been explained by the inactivation of the bacterial proteins and enzymes by interacting with the constituent thiol groups and by the inactivation of the deoxyribonucleic acid (DNA) replication due to the interaction of the ionic silver with the negatively charged phosphorus atoms in the bacterial DNA [39,40]. The inactivation of the DNA inhibits the replication of the microbes (Figure 9). Interestingly, even though the silver nanoparticles and ions are antimicrobial in action, they are considered completely safe for human use. This can be explained by the non-toxic nature of the silver nanoparticles and ions even when it is used in relatively high concentrations [38]. In [38], the researchers synthesized chitosan and silver nanoparticle-based nanocomposites to enhance food quality and safety. For this purpose, the researchers dissolved chitosan flakes in lactic acid solution at 70 °C for 1 h. The filtered solution was poured into polypropylene Petri plates and dried at 60 °C under ambient humidity conditions. This method of developing films is generally regarded as the solution casting method (Figure 10). The films were then harvested from the Petri plates by dipping the Petri plates in 1 M sodium hydroxide solution. The films were then thoroughly washed with distilled water and dried. The dried films were then soaked in silver nitrate solution for 12 h, followed by soaking trisodium citrate solution for another 12 h. The treatment with the trisodium citrate solution resulted in the conversion of the silver ions from silver nitrate, which diffused into the polymer matrices, to silver nanoparticles. The resultant films were then dried to obtain nanocomposite films of chitosan and silver nanoparticles. The films were then thoroughly characterized by transmission electron microscopy, X-ray diffraction, Fourier transform infrared spectroscopy, and thermogravimetric analysis methods. The analysis of the characterization results helped to establish the presence of silver nanoparticles within the nanocomposite films. The size of the formed silver nanoparticles varied in the range of 75 nm and 250 nm, respectively. The prepared nanocomposite films showed excellent antimicrobial properties against *E. coli*. The authors concluded that the proposed methodology could be employed to develop the silver nanoparticle/chitosan nanocomposite films on a large scale for industrial applications. In another study, *Nigella sativa* extract was used to synthesize biogenic silver nanoparticles, which exhibited antioxidant activity [41].

**Figure 11:**
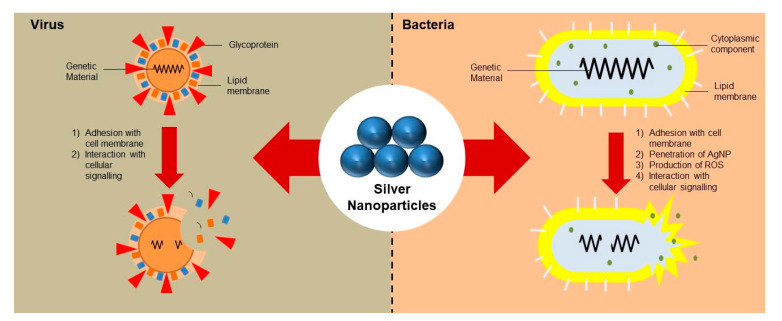
Overview of mechanism of antimicrobial activity of silver nanoparticles. (Reproduced from [46]).

The biogenic silver nanoparticles were used for the development of the chitosan/silver nanoparticle-based nanocomposites. One of the major challenges in developing silver nanoparticle-based nanocomposites is the leakage of the nanoparticles. Accordingly, Wu et al. (2018) proposed the immobilization of the silver nanoparticles on laponite, a synthetic clay that appears as nanodisks [44]. The immobilization of the silver nanoparticles on the laponite nanodisk was carried out by the reactive template grain growth method. The composite films were prepared by the solution casting method. Subsequently, the films were neutralized by 5% (*w/v*) sodium hydroxide solution followed by washing with water. Finally, the films were dried in a controlled environment. The prepared films were used for the preservation of litchis. It was found that the proposed packaging materials were able to protect the litchis better than the commercially employed cling wrap. In a similar study by Gu et al. (2021), silver nanoparticles were immobilized over reduced graphene oxide [45]. A layer of immobilized silver nanoparticles was sandwiched in between two layers of chitosan. Initially, a chitosan film was formed by solution casting, followed by the addition of a suspension of immobilized silver nanoparticles to form a layer of immobilized silver nanoparticles, and followed by another chitosan film. The proposed films showed antimicrobial activity for prolonged periods (Figure 11).

Blends of polymers have been explored to synthesize nanocomposites for food packaging applications. The blending of polymers allows improving the properties over the polymer matrices that consist of a single polymer. Accordingly, in [47], chitosan/gelatin/silver nanoparticle-based nanocomposites were prepared. For the preparation of the chitosan, a 2% (*w*/*v*) chitosan solution was prepared in 2% (*v*/*v*) acetic acid solution. Separately, a 2% (*w*/*v*) gelatin solution containing previously synthesized silver nanoparticles was prepared. Then, 90 mL of the chitosan solution and the colloidal dispersion in gelatin solution were mixed and homogenized thoroughly.

**Figure 12:**
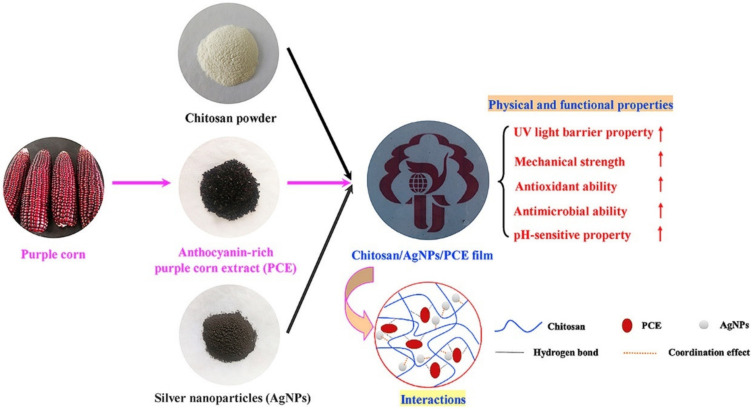
Antioxidant, antimicrobial, and pH-sensitive films based on chitosan, silver nanoparticles, and purple corn extract. (Reproduced with permission from [48]).

Polyethylene glycol was used as the plasticizer, which could impart flexibility to the prepared films. The liquid mixtures were then converted into films by the solution casting method. The extract of the fruits of *Mimuosops elengi* was used to synthesize silver nanoparticles. The film without the silver nanoparticles was considered as the control. The incorporation of the silver nanoparticles reduced the transparency, which can help prevent photodegradation of the food products and enhance the mechanical properties. The films could greatly enhance the shelf life of the red grapes. In a recent study, silver nanoparticles and anthocyanin-rich purple corn extract were used to develop intelligent, active packaging systems (Figure 12) [48]. The films exhibited not only antimicrobial properties but also exhibited antioxidant and pH-sensitive properties. The pH-sensitive property, which was due to the purple corn extract, may be explored in detecting spoilage of the food products. For the development of the films, silver nanoparticles, purple corn extract, or a combination of these were added to the chitosan solution in acetic acid solution to develop the film-forming mixture. The films were developed by the solvent casting method. The films of chitosan containing both silver nanoparticles and purple corn extract showed better UV-blocking and mechanical properties.

### 3.2. Chitosan and Cellulose-Based Nanostructure Containing Food Packaging Systems

**Figure 13:**
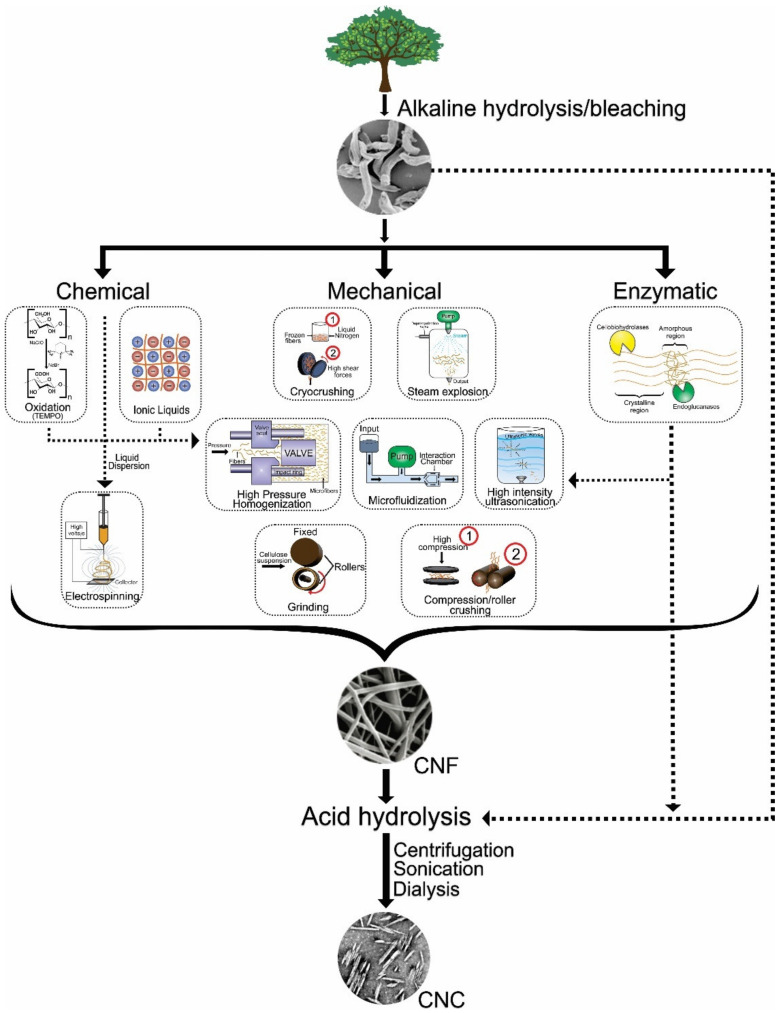
Conventional methods to synthesize cellulose nanoparticles. (Reproduced from [57]).

Cellulose is the most widely available biopolymer in nature. It is biocompatible and well-tolerated by the human body. Hence, cellulose-based products have been used in the food and pharmaceutical industries for a long time. Various nanostructures have been developed using cellulose as the base material. Some of the nanostructures include nanocrystals, nanoparticles, and nano-whisker [49]. The conventional methods of cellulose nanoparticle synthesis are summarized in Figure 13. The inclusion of cellulose-based nanostructures has been employed to improve the mechanical properties of the films and coatings. Further, cellulose nanostructures can help to reduce the water vapor transmission rate. The improvement in the properties of the chitosan films due to the addition of cellulose nanostructures can be related to increased hydrogen bonding that consequently increases the crystallinity of the chitosan films [50]. In [51], cellulose nanocrystal and chitosan-based nanocomposites were prepared by the solution casting method. The addition of cellulose nanocrystals at a concentration of 4% significantly improved the mechanical properties of the chitosan films. The addition of the cellulose nanocrystal reduced the water solubility, moisture absorption, and water vapor permeation of the chitosan films. The UV light barrier properties of the chitosan films were also improved upon the addition of the cellulose nanocrystals. The authors concluded the possibility of using the cellulose nanocrystal-loaded chitosan films for developing sustainable packaging material for food applications. The improved mechanical property of chitosan films with the addition of cellulose nanocrystals is also associated with a reduction in ductility. Similar observations have also been made in chitosan/guar gum/nanocrystalline cellulose nanocomposite films [52]. However, the addition of cellulose nanocrystals in concentrations greater than 8% can severely reduce the strength and ductility of the chitosan films [53]. The effect of cellulose nanocrystals, obtained from different sources (coconut shell, corn husk, and corncob), on the properties of chitosan/glycerol films, were studied [54]. The observations from the study suggested that the source of nanocrystals plays a significant role in governing the mechanical properties of the chitosan/glycerol films. The addition of grape pomace extract, which is rich in phenolic compounds, in the chitosan/cellulose nanocrystal nanocomposites, can consequently increase the antioxidant properties of the nanocomposites [55]. The inclusion of the extract within the nanocomposite films hampered the mechanical strength of the films. However, the extract could help to increase the ductility of the films. The curing of chitosan films is an effective method for improving the mechanical properties. This can be achieved either by conventional heating method or by microwave irradiation [56]. It has been found that the employment of microwave irradiation for curing is a better method than the conventional heat curing method. It was found that the microwave-cured films demonstrated considerably lowered solubility, swelling, and water vapor permeability over the heat-cured films.

Salari et al. (2018) reported the synthesis of chitosan nanocomposite films with two nanostructures types, namely, bacterial cellulose nanocrystals and silver nanoparticles [58]. Both the nanostructures improved the water-resistance of the films. However, the bacterial cellulose nanocrystals could enhance the mechanical and thermal stabilities of the films. On the other hand, silver nanoparticles were mainly responsible for enhancing the antimicrobial properties of the films against foodborne pathogens. The chitosan nanocomposite films containing both the nanostructures were found to be suitable for food packaging applications. In a similar experiment, dual nanosystems (cellulose nanowhiskers and multiwall carbon nanotubes) were used to improve the properties of the chitosan films [59]. Two types of multiwall carbon nanotubes were employed, viz., functionalized and non-functionalized. It was found that the nanocomposites of chitosan/cellulose nanowhiskers and functionalized multiwall carbon nanotubes were highly stable. The authors reported that the developed nanocomposite films could be exploited for food packaging applications.

In the other study, cellulose nanocrystals were used to develop a Pickering emulsion of oleic acid [60]. The cellulose nanocrystals acted as the stabilizer of the emulsion. The external phase of the emulsion was then added with chitosan. The chitosan-based Pickering emulsion was used as the coating solution for Bartlett pears. This resulted in the formation of hydrophobized films over the Bartlett pears. The coatings were found to delay the ripening of the fruit for a prolonged duration. The developed coating was more efficient than the commercially available coating material, Semperfresh™. The study was conducted for 3 months. The authors concluded that hydrophobization of the chitosan-based hydrophilic coating materials could significantly enhance the shelf life of fruits.

Adel et al. (2019) synthesized a ternary nanocomposite that contained oxidized nanocellulose and β-cyclodextrin citrate within the matrix of chitosan [61]. The Clove essential oil was incorporated within the films to enhance the antimicrobial and antioxidant properties of the films. The films were prepared by the solution casting method. The oxidized nanocellulose helped to improve the mechanical properties of the films. Further, the water vapor permeability through the films was also constrained by the addition of oxidized nanocellulose. The rate of release of the clove essential oil was prolonged by adding oxidized nanocellulose within the films.

Cellulose nanofibers can also be prepared easily by the electrospinning method. These nanofibers have been explored for developing nanocomposites for food packaging applications. In [62], the authors prepared the nanocomposite of chitosan and cellulose nanofibers. The nanocomposite films were prepared by the solution casting method. The nanocomposite films also contained glycerol. Glycerol was found to decrease the solubility of the films and promote the formation of the quasi-elastomeric structure. The cellulose nanofibers improved the barrier and mechanical properties of the chitosan nanocomposite films. These nanocomposite films were proposed for food packaging applications.

### 3.3. Chitosan and Montmorillonite Nanoclay Containing Food Packaging Systems

**Figure 14:**
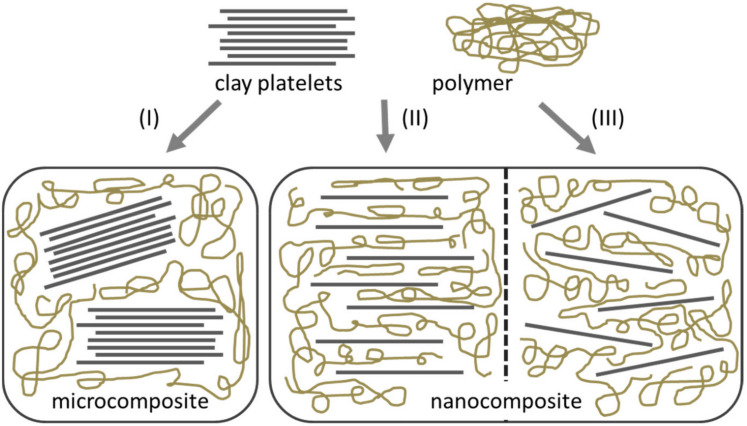
Overview of mechanism of formation of nanoclay-based nanocomposite. (**I**) Phase-separated microcomposite, (**II**) intercalated nanocomposites, and (**III**) exfoliated nanocomposites. (Reproduced from [70]).

Montmorillonite is a mineral clay [63]. Chemically, it is a hydrated, alumina–silicate layered clay [64]. It has been extensively used as a reinforcing agent for food packaging material (Figure 14). Additionally, it has other important properties like swelling capability and plasticizing effect, for which it is one of the most sought-after clays for designing food packaging materials [63]. Chitosan/montmorillonite composite coatings were used to develop an oil-resistant paper for food packaging applications [65]. The developed packaging material also exhibited lower air permeability. It was also found that the coating increased the mechanical properties and tearing resistance of the packaging paper compared to the uncoated packaging paper. This was attributed to the filling of montmorillonite into the network of the cellulosic fibers. In a similar experiment, chitosan/ thiabendazolium-modified montmorillonite coating materials were developed. The coated date palm fiber trays were coated with chitosan/modified montmorillonite, forming a bilayer material. The coating not only increased the mechanical properties of the trays and hydrophobicity of the trays but also showed good inhibitory activity against several microbes like *Escherichia coli*, *Staphylococcus aureus*, and *Pseudomonas aeruginosa*. In [63], rosemary essential oil-loaded nanocomposites of chitosan and commercially available montmorillonite nanoclays (e.g., Cloisite^®®^Na^+^, Cloisite^®®^Ca^++^, and Cloisite^®®^20) were developed. The addition of the nanoclay improved the mechanical properties of the chitosan films due to the reinforcing property. The loading of rosemary essential oil compromised the mechanical property of the nanocomposite films. Further, the water solubility of the nanocomposite films was increased upon the addition of the essential oil. However, the UV light blocking property was improved. In general, the rosemary essential oil-loaded chitosan nanocomposite films showed sufficient properties to be explored for food packaging applications. In a similar study, the chitosan nanocomposites that contained montmorillonite nanoclays (Cloisite^®®^Na^+^, and Cloisite^®®^Ca^++^) as the reinforcing agent were prepared [66]. The nanocomposite films were loaded with rosemary essential oil (REO) or ginger essential oil. The films were tested as active food packaging systems for the storage of fresh poultry meat, which were kept under refrigeration for 15 days. The nanoclays were reported to extensively prevent lipid oxidation and microbial contamination of the meat. The inclusion of the essential oils improved the antioxidant properties of the nanocomposite films. A similar study on ginger essential oil-loaded chitosan/ montmorillonite nanoclay nanocomposite was reported in [67]. The nanocomposite films showed better mechanical and barrier properties against water vapor and UV light. The developed nanocomposite films were proposed for enhancing the shelf life of food products with high unsaturated fat content. Cui et al. (2020) reported the loading of cinnamaldehyde in acidified modified montmorillonite. The cinnamaldehyde-loaded modified clay was consequently used as a reinforcing agent for the chitosan matrices [68]. The nanocomposite films showed better UV resistance, water vapor barrier, rigidity, and ductility. The prepared films also showed excellent antimicrobial properties against the microbes *Staphylococcus aureus* and *Escherichia coli*. These films were reported to have sufficient properties for food packaging applications. It is interesting to note that while developing chitosan/montmorillonite/essential oil films, the addition of montmorillonite at the initial stage results in the formation of films with higher mechanical properties [69]. This was ascribed to the exfoliation of the montmorillonite when it is added at the initial stage that promotes the formation of a compact nanocomposite structure. On the contrary, an intercalated structure is formed when the montmorillonite is added at a later stage.

Another study reported organically modified sodium montmorillonite, and zinc oxide nanoparticles were incorporated within the chitosan films [71]. The distribution of zinc oxide nanoparticles in the presence of modified sodium montmorillonite was relatively homogenous. This suggested that the modified sodium montmorillonite improved the compatibility of the zinc oxide nanoparticles and the chitosan polymer chains. The thermal stability of the films was not much affected by the modified sodium montmorillonite. However, the same was greatly affected when zinc oxide nanoparticles were incorporated within the films. Synergistic antimicrobial activity of chitosan and zinc oxide nanoparticles was observed in the composite films. Such dual-reinforcement nanocomposite films were proposed for developing novel food packaging systems. In another study, α-tocopherol-chitosan nanoparticles were incorporated within the chitosan/montmorillonite nanocomposite films [72]. The films were tested to enhance the shelf life of sliced dry-cured ham for 120 days at 4 °C. The study revealed that the films could retain their antioxidant properties even after 16 days of storage.

The montmorillonite nanoclay has also been incorporated within chitosan/polyvinyl alcohol matrices [73]. Sodium lactate was incorporated within the films to impart antimicrobial activity to the films. Intermolecular hydrogen bonding among the montmorillonite nanoclay, chitosan, and polyvinyl alcohol played an important role during the formation of the nanocomposite films. The addition of montmorillonite nanoclay at a concentration of ≤15% significantly improved the tensile strength of the nanocomposite films. The Fickain-diffusion equation could well explain the release of sodium lactate from the films. The diffusion of the sodium lactate from the films was reported to be pH-dependent. The properties of the nanocomposite films suggested that the nanocomposite films could be tried for food packaging applications.

A summary of the chitosan-based nanocomposites is compiled in Table 1.

## 4. Starch-Based Nanocomposites

Starch is one of the commonly available polysaccharides present in vegetables, fruits, cereals, etc. [74]. Chemically, starch has been described as a polymer of glucose molecules. These glucose molecules are linked together through the glycosidic linkages [75]. There are two sub-units of starches, namely, amylose and amylopectin. The ratios of these sub-units vary in starches obtained from different sources (e.g., corn starch, potato starch, and lotus starch). It appears in granular form. The shape of the starch granules may be spherical, oval, or irregular, whose diameter may vary in the range of 0.1–200 μm [75]. Starch is considered a semi-crystalline polymer, which can account for the existence of a significantly greater extent of hydrogen bonding. Due to this reason, the starch granules are insoluble in cold water. This can be explained by maintaining the structural integrity of the starch granules, which have a compact structure. The compact structure of the starch granules makes them resistant to water ingression. However, when the starch granule suspension is heated, the absorption of water molecules is promoted, which in turn results in the expansion of the compact structures of the starch granules [76]. This phenomenon promotes the leaching of the amylose sub-units from the starch granules. Consequent heating of the suspension leads to the total structural disintegration of the granule structure, leading to the formation of a colloidal solution. The process of formation of the colloidal suspension upon heating of the starch granule suspension is regarded as gelatinization. A consequent cooling results in the formation of gel, regarded as the retrogradation of starch. The process of retrogradation is promoted by the molecular reorganization and the re-establishment of the hydrogen bonds within the polymeric chains. These phenomena help to reestablish the ordered structures within the hydrated starch molecules, which helps to induce gelation.

Since starch is a naturally derived polysaccharide, it is available at a low price. The polymer is also biodegradable and non-toxic for human consumption. Due to these reasons, researchers have extensively explored the use of starch for developing edible films and coatings for food packaging applications [77]. The properties of starch films have been improved by blending with other polymers, reinforcing with different reinforcing materials, and even by crosslinking [78,79]. Many researchers have proposed physical and chemical modifications of the starches for improving the properties of the films and coatings [80]. Bioactive and intelligent films and coatings of starch have also been proposed for food packaging applications.

### 4.1. Starch and Silver Nanoparticle-Containing Food Packaging Systems

Jung et al. (2018) reported the synthesis of a starch-based solution that contained silver nanoparticles as the coating material for the paper [81]. The solution was synthesized by one-step synthesis wherein ultrasonication was employed. In the study, a mixture of starch, silver nitrate, and water was prepared, followed by the ultrasonication of the mixture for 60 min at 90 °C. This process led to the in situ formation of the silver nanoparticles, which resulted in the conversion of the colorless mixture into a dark red solution. Thereafter, the solution was cooled down to 25 °C, which was then used for the coating of the paper for food packaging applications. The coating of the papers helped to improve the oil resistance of the paper. Further, the coated papers showed sufficient antimicrobial properties to be used for food packaging applications. A similar study was reported by Dang et al. (2018), where the authors reported the development of a coating mixture by combining hydrophobic noncrystalline porous starch (a microporous starch) and silver nanoparticles [82]. The coating mixture was used to coat the paper, which improved the oil resistance and the mechanical properties of the packaging paper. The coated paper exhibited good antimicrobial properties against *Escherichia coli* and *Staphylococcus aureus*. The authors reported that the coated papers could be explored for food packaging applications. Sugar palm starch/silver nanocomposites were proposed for antibacterial coating applications in [83]. The thermal stability of the nanocomposites was higher than the pristine starch film. Amazonian tuber starch and silver nanoparticles-based nanocomposite coatings were developed in [84]. Camu-camu fruit was coated with starch/silver nanocomposite coatings. The coatings were able to delay the ripening of the fruits and consequently enhanced their shelf life.

Apart from starch/silver nanocomposite coatings, starch/silver nanocomposite films have also been proposed for food packaging applications. In [85], starch/silver nanocomposites were prepared. The silver nanoparticles were synthesized by the green synthesis method. Silver nanoparticles were incorporated in the films at low concentrations so that the prepared films did not elicit any cytotoxic effects. The incorporation of the nanoparticles increased the thickness and opacity of the starch films and imparted antimicrobial activity against foodborne pathogens (*E. coli* and *Salmonella* spp.). The silver nanoparticle-loaded films were able to extend the shelf life of fresh cheese samples for 21 days. In a similar study, Mohseni et al. (2020) synthesized silver nanoparticles by the green method using pomegranate seeds extract [86]. The developed films were found to be suitable for food packaging applications. The synthesis of starch/silver nanocomposite films for food packaging applications was proposed by the extrusion and compression molding method [87]. The films were able to delay the growth of *E. coli* and were proposed for biodegradable food packaging applications. In [88], lemon juice was used as the reducing and stabilizing agent for the green synthesis of silver nanoparticles. The nanoparticles were used to develop starch nanocomposite films. In a separate study, guava leaves extract solution, obtained from the discarded leaves, were incorporated within corn starch/silver nanocomposites [89]. The films showed good UV light-blocking properties. The antibacterial activity of the films against *S. aureus* was better as compared to *P. aeruginosa*. The films had sufficient properties for food packaging applications.

In an interesting study, starch-based nanocomposite films were prepared, where multiple nanoparticles, either singly or in combinations, were used for the reinforcement [90]. The nanocomposites were prepared using silver nanoparticles, zinc oxide nanoparticles, and copper oxide nanoparticles. The preparation of the films was achieved by the solution casting method. The silver and zinc oxide nanoparticle-loaded composites showed the highest antimicrobial activity. The efficacy of the nanocomposites in retarding the growth of microbes was studied against *S. aureus* and *E. coli*. An increase in the nanoparticle content increased the antimicrobial activity of the nanocomposite films. The amount of the nanoparticles varied in the range of 1% (*w*/*w*) and 3% (*w*/*w*). The water vapor transmission rate of the nanocomposite films was lower than the pristine starch films.

Starch blends have been explored for designing nanocomposite-based food packaging material. In one such study, cassava starch/agar blends were used to develop silver and zinc oxide-containing nanocomposite films [91]. The films were synthesized by the solution casting method. Incorporation of the nanoparticles reduced the water solubility, moisture content, and water vapor permeability of the cassava starch/agar blend films. The antimicrobial activity study suggested a substantial reduction in the growth kinetics of *Pseudomonas aeruginosa* and *Staphylococcus aureus*. The films were proposed for food packaging applications. In another study, polyvinyl alcohol and boiled rice starch blend film loaded with silver nanoparticles were proposed for food packaging applications [92]. The films were synthesized in situ by the one-step synthesis method under sunlight irradiation. The color of the nanocomposite films was dark brown. It was found that the components of the films had physical interactions, which helped to improve the mechanical properties of the nanocomposite film over the polyvinyl alcohol-only control film. The optical and antimicrobial properties of the nanocomposite films were also superior to the control film. the overall properties of the nanocomposite films were sufficient to be explored for food packaging applications. The inclusion of oregano essential oil in polyvinyl alcohol/cassava starch/silver nanocomposites was proposed in [93]. The combination of oregano essential oil and silver nanoparticles exhibited a synergistic antimicrobial activity.

### 4.2. Starch and Cellulose-Based Nanostructure Containing Food Packaging Systems

Starch-based films have gained attention for food packaging applications. However, these films suffer from poor water resistance and mechanical properties. To improve these properties of the starch films, Jeevahan et al. (2019) reported the reinforcement of the native rice starch films with nanocellulose [94]. The nanocellulose, which was used as the reinforcement agent in the concentration range of 0–10%, was synthesized from banana pseudostems. It was found that the addition of nanocellulose reduced the water vapor permeability and increased the films’ mechanical stability. The films showed good promise to be utilized as food packaging material.

In [95], cellulose nanofibers were used as the reinforcing agent for the glycerol-plasticized starch films. The cellulose nanofibers were synthesized from short henequen fibers (*Agave fourcroydes*) via the chemo-mechanical process. The addition of cellulose nanofibers at a concentration of 0.4% (*w*/*w*) markedly improved the mechanical properties of the starch films. A further increase in the cellulose nanofiber content decreased the mechanical stability of the films. Yuan et al. (2021) reported the synthesis of starch nanocomposite films, which contained silver nanoparticle immobilized cellulose nanofibrils as the reinforcing agent [96]. The reinforcement of the starch films with the hybrid nanofibrils enhanced the mechanical stability and the antimicrobial property of the nanocomposite films. The antimicrobial property of the films was tested against *Escherichia coli* and *Staphylococcus aureus*. The film with 5% of the reinforcing agent exhibited the most enhanced mechanical properties. The nanocomposite films also exhibited better barrier properties and thermal stability over the starch-only films. The films were biocompatible and could be tried for food packaging applications. Cellulose nanofibers from the carrot pulp have been proposed for the development of starch nanocomposite films [97]. The nanocomposite films were then loaded with *Eucalyptus globulus* leaf extract. Such films were found to exhibit better barrier and antioxidant properties. It was found that these films were able to enhance the shelf life of grapes, which were stored at room temperature (25 ± 2 °C) and under refrigeration (4 ± 1 °C). At room temperature, the films were able to protect the fruit for 7 days while it was 28 days under refrigeration. The films were also tested for their ability to prevent microbial spoilage. It was observed that the extract-loaded films were capable of hindering the growth of *Escherichia coli*, *Listeria monocytogenes*, *Salmonella typhimurium*, and *Penicillium* spp. The authors reported that the developed nanocomposite films were suitable for developing active food packaging systems. Sugar palm (*Arenga pinnata*) starch-based nanocomposites were developed using sugar palm nanofibrillated celluloses as the reinforcing agent [98]. Interestingly, both sugar palm starch and sugar palm nano-fibrillated celluloses are considered agro wastes. The nanocomposite films were developed by the solution casting method. The amount of nano-fibrillated celluloses varied from 0.1% to 1.0% (*w*/*w*) in the nanocomposites. The nanocomposite films exhibited enhanced water resistance and improved thermal stability. This was associated with the strong hydrogen bonding among both the continuum and dispersed phases. Since both the phases were biodegradable, the authors reported that the developed nanocomposite films could be explored for biodegradable packaging applications. A similar study on cassava bagasse cellulose nanofibrils and cassava starch nanocomposites was reported in [99]. In this study, either glycerol or a mixture of glycerol/sorbitol was used as a plasticizer for developing a thermoplastic cassava starch matrix.

Cellulose nanocrystallites (nanocrystals) synthesized from cotton linters by the sonochemical method have been proposed to reinforce glycerol-plasticized thermoplastic starch [100]. The nanocomposite films were developed by the solution casting method. The thermal stability of the films was studied by thermogravimetric analysis under the nitrogen environment. It was found that the cellulose nanocrystals significantly affected the activation energy for the thermal degradation of the nanocomposite films. The developed films were proposed for applications in food packaging. Sugar palm nanocrystalline cellulose, derived from sugar palm fibers, has been used as the reinforcing agent for developing nanocomposites of sugar palm starch [101]. The reinforcement of the starch matrix helped to improve the mechanical and thermal properties. The enhancement in the properties of the starch matrix accounted for the enhanced crystallinity of the nanocomposites. The optimum concentration of the nanofiller was found to be 0.5% (*w*/*w*). In [102], bacteriocins that were extracted from the cultures of *P. acidilactici* and *E. faecium* were immobilized over the surface of the cellulose nanocrystals. The bacteriocin-immobilized cellulose nanocrystals were used for the reinforcement of the starch films. The films containing bacteriocin remained fresh for 28 days. On the contrary, the films without the bacteriocin started degradation due to fungal growth after 14 days.

Corn starch and bacterial cellulose nanowhiskers-based nanocomposite films were prepared by the melt blending process in [103]. The nanocomposite films showed significant alteration in the morphology, mechanical properties, and optical and barrier properties as compared to the corn starch films. The optimal barrier properties could be achieved at the bacterial cellulose nanowhiskers concentration of 15% (*w*/*w*). The optimized nanocomposite films were then coated either with the electrospun poly(3-hydroxybutyrate) nanofibers or electrospun poly(3-hydroxybutyrate)/bacterial cellulose nanowhiskers nanofibers. Such films showed good barrier properties. The authors suggested that such multi-layered films can be explored for food packaging applications.

### 4.3. Starch and Montmorillonite Nanoclay Containing Food Packaging Systems

Iranian potato starch and montmorillonite containing nanocomposite were prepared in [104]. The amount of montmorillonite within the nanocomposite was varied up to 5% (*w*/*w*). Glycerol was used as the plasticizer for the synthesized nanocomposites. The addition of 5% nanoclay improved the thermal stability of the films. Also, there were significant changes in the color parameters with the addition of the nanoclay. It was interesting to note that the addition of the nanoclay improved the UV-blocking properties of the films without compromising their opacity in the visible region. The roughness of the plasticized films was lower as compared to the non-plasticized films. In [105], nanocomposites of cationic starch and montmorillonite nanoclay were developed. It was found that 3% montmorillonite in the cationic starch matrices was exfoliated. The exfoliation of the nanoclay was confirmed by XRD studies. Further, it was found that there was an establishment of new hydrogen bondings. The new hydrogen bondings were established among the hydroxyl groups of the cationic starch and the nanoclays. The incorporation of montmorillonite improved the tensile strength of the nanocomposite films. However, the ductility of the films was lost. The nanocomposite films exhibited good barrier and optical properties that make them suitable for food packaging applications.

De Souza et al. (2020) incorporated carvacrol essential oil and montmorillonite within the starch films [106]. The thermal stability of the films was improved over the starch-only film. This observation accounted for the formation of hydrogen bonding among the starch molecules and montmorillonite. The biocidal activity of the films against *E. coli* was due to the synergistic activity of montmorillonite and carvacrol. The developed films were proposed for developing antimicrobial packaging materials for fresh foods, including fruits and vegetables. In a similar study, Campos-Requena et al. (2017) synthesized thermoplastic starch and montmorillonite-based nanocomposite films [107]. The films were loaded with the single components of essential oils, namely, carvacrol and thymol. Synthesis of the nanocomposite films was carried out by the extrusion method. Improved mechanical and thermal properties were observed, which accounted for montmorillonite’s presence in the intercalated/exfoliated morphology. It was observed that the nanocomposite films could sustain the release of the single components of essential oils, which would help extend the shelf life of the food products. Accordingly, the ability of the nanocomposite films as food packaging materials was tested using strawberries, which were inoculated with the spoilage mold, *Botrytis cinerea*. The use of the nanocomposite films helped to increase the shelf life without compromising the fruit quality and the organoleptic properties.

Polymeric blends of cassava starch and polycaprolactone were reinforced with sodium montmorillonite [108]. Synthesis of the blends was achieved by the extrusion process. However, the films were developed by compression molding. Glycerol was used as the plasticizer for the composite films. The addition of polycaprolactone and montmorillonite compromised the solubility of the films and consequently improved the barrier properties. The composite films were proposed for the synthesis of packaging materials for flours and bakery products. Kumar et al. (2018) proposed using a laboratory food extruder to synthesize the starch/montmorillonite nanocomposite [109]. The authors reported that exfoliation of the nanoclay within the starch/nanoclay blend was achieved during the extrusion process. Thereafter, sodium caseinate was blended with the starch/nanoclay blend, which was then used for the synthesis of the nanocomposite films. The addition of the nanoclay into the polymer blend significantly affected the mechanical, water sorption, and solubility properties of the nanocomposite films. The authors had concluded that it is possible to exfoliate the nanoclay using a laboratory food extruder. The authors also stated that though the films have shown great promise for food packaging applications. However, further experiments are needed to establish the food safety of the prepared nanocomposite films.

Ternary nanocomposite films of crosslinked wheat starch, sodium montmorillonite, and titanium dioxide nanoparticles were proposed in [110]. The synthesis of the films was carried out using the solution casting method. In this study, the wheat starch was crosslinked with sodium trimetaphosphate and sodium tripolyphosphate. The films prepared with the crosslinked wheat starch showed better mechanical and thermal properties than the films synthesized using the native wheat starch. However, a reduction in ductility was observed with the addition of the nanoclay. The mechanical and thermal properties improvement can be explained by the formation of new hydrogen bonds among the crosslinked starch and nanoparticles.

Mathew et al. (2018) proposed the synthesis of silver nanoparticles in the presence of boiled rice water, which contains starch in abundance [111]. The boiled rice water was used as the reducing agent for the in situ synthesis of silver nanoparticles, which was achieved under sunlight. The solution casting method was used to develop silver nanoparticle reinforced montmorillonite/poly(vinyl alcohol)/boiled rice starch nanocomposite films. The produced films were found to have suitable properties for food packaging applications.

A summary of the starch-based nanocomposites is compiled in Table 2.

## 5. Cellulose-Based Nanocomposites

**Figure 15:**
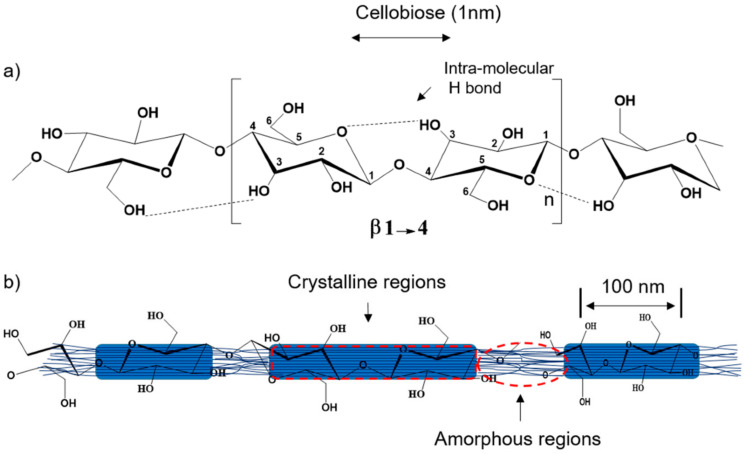
(**a**) Schematic of cellulose repeating unit with the β-(1,4)-glycosidic linkage, dotted lines indicate intramolecular hydrogen bond; (**b**) hypothetical configuration of ordered (crystalline) and disordered (amorphous) regions in cellulose nanofibrils. (Reproduced from [112]).

Celluloses are abundant in nature and can be easily extracted from the cell walls of plants. Since the polymer is abundantly present in nature, the cost for the procurement of the cellulose as the raw material is very economical. It can be extracted from agricultural wastes (e.g., fruits, vegetables, rice stalk, and cotton stalk) and other forest residues. The wastes generated from the food processing industries (e.g., mango peel and sugarcane bagasse) can also be used to obtain celluloses. Chemically, cellulose consists of β-d-glucopyranose monomers that are linked by β-1,4-glycosidic linkages (Figure 15). It is categorized as a linear polysaccharide. The polymer is highly biocompatible and biodegradable. The polymer is also categorized as edible polymers, i.e., cellulose is safe for human consumption. Additionally, cellulose films have shown an excellent capability to screen UV radiations. Due to these reasons, it has been extensively used by the pharmaceutical, biomedical, and food industries to develop different food products and food packaging systems. The food packaging systems have been developed with cellulose and cellulose derivative (e.g., cellulose acetate, cellulose sulfate, and carboxymethyl cellulose)-based products with great success. Cellulose-based food packaging systems are capable of entrapping and consequently releasing essential oils, which exhibit outstanding antimicrobial and antioxidant properties. Such packaging systems have played a crucial role in preventing food wastage due to ripening and microbial spoilage. The polymeric architectures developed by the cellulosic materials offer good mechanical and thermal properties as compared to the other polysaccharide-based architectures. The celluloses have also been explored to develop nanosystems like cellulose nanocrystals, nano cellulose, etc., as the reinforcing agent for the development of food packaging systems. In this regard, cellulosic fibers have also been explored as reinforcing agents. The use of cellulosic materials has allowed the industries to minimize the use of synthetic non-biodegradable polymers for food packaging systems. However, it is important to note that the complete elimination of the non-biodegradable polymers is not possible at this stage.

In recent years, the interest in bacterial cellulose has gained much attention in various industries, including food, due to its extraordinary properties. Conventionally, bacterial cellulose has been detected in multiple traditional foods (e.g., kombucha tea) of Asian countries. The chemical identity of the bacterial cellulose is the same as that of the cellulose obtained from vegetables [113,114,115]. However, the bacterial cellulose is purer than the cellulose that is from vegetable sources, which often also consists of other components like lignin, hemicellulose, or pectin. The cellulose is secreted as an exopolysaccharide by many aerobic bacteria, e.g., *Achromobacter*, *Aerobacter*, *Azotobacter*, *Alcaligenes*, *Komagataeibacter*, *Agrobacterium*, etc. In this section, the different types of cellulose-based nanocomposite materials for food packaging applications will be discussed. The advantages and the properties of such materials will also be discussed herein.

### 5.1. Cellulose and Silver Nanoparticle-Containing Food Packaging Systems

As an example, nanocomposite films containing silver nanoparticles and cellulose were synthesized in [116]. In the study, the authors had initially synthesized silver nanoparticles by the in situ silver nitrate reduction method wherein N, N-dimethylacetamide was used as a reducing agent and poly (vinyl pyrrolidone)-K30 as the stabilizer. Lithium chloride was added to the colloidal dispersion of the silver nanoparticles. The addition of the lithium chloride helped to solubilize cellulose by diminishing the hydrogen bonds in cellulose. Thereafter, the silver nanoparticles and the cellulose were conjugated using γ-mercaptopropyltrimethoxysilane. The mixtures were then converted into nanocomposite films by the solution casting method. The prepared films exhibited excellent mechanical, thermal, and optical transparency properties. They also exhibited excellent antimicrobial activity against *Staphylococcus aureus* and *Escherichia coli*. The films were proposed for food packaging applications. In an analogous study, bacterial cellulose/silver nanoparticle composites were prepared for food packaging applications [117]. The nanocomposite films provided better protection against microbial spoilage as compared to the silver nanoparticle colloidal dispersion. The reduced activity of the silver nanoparticle colloidal dispersion was attributed to the agglomeration of the silver nanoparticles within the colloidal dispersion. The composite films were able to enhance the shelf life of the freshly harvested tomatoes to 30 days as against the shelf life of 15 days when polyethylene bags were used. In [118], the authors proposed synthesizing hyperbranched polyamide-amine conjugated silver nanoparticles over the oxidized cellulose, which was successively used to develop regenerated cellulose films. These films showed low leakage of the silver nanoparticles without compromising the antimicrobial activity of the films against *Escherichia coli* and *Staphylococcus aureus*.

The nanocomposite films of cellulose acetate and silver nanoparticles were prepared in [119]. The silver nanoparticles were synthesized using various poly-phenols (e.g., gallic acid, pyrogallic acid, quercetin, and rutin), which helped to synthesize environmentally safe nanocomposites. The nanocomposite films were synthesized by the solution casting technique. Though the films showed good antibacterial properties, the antibacterial activity was differential. It was found that the activity was higher in *St. aureus*, *B. cereus*, *S. typhi*, *E. coli*, and *K. pneumonia* in comparison to the two strains of *Pseudomonase spp*. The toxicity was found to be low against larvae where the mortality was lower than 18%. Further, the leaching of the silver nanoparticles was much lower than the acceptable limit. It was concluded that the developed films were suitable for active food packaging applications.

Nanocomposites of cellulose nanofibril and silver nanoparticles have been developed for food packaging applications [120]. In situ synthesis of the silver nanoparticles within the cellulose nanofibrils was carried out to develop nanocomposites. The nanocomposites were then used to develop films by the solution casting method. The properties and the cytotoxicity of the nanocomposites were then tested. It was observed that the silver nanoparticles were homogeneously distributed within the composites after the in situ synthesis. However, the silver nanoparticles exhibited agglomeration in the films. The formation of silver nanoparticles and cellulose nanofibril-based nanocomposites weakened the hydrogen bonding of the cellulose. It was observed that the silver nanoparticles were released from the composite films during the initial 24 h, which were responsible for the antimicrobial activity of the composite films against *Escherichia coli* and *Listeria monocytogenes*. The synthesized nanocomposite films were found to be non-cytotoxic even though the silver nanoparticles were observed to be taken up by the colon cells by the endosomal mechanism. Such nanocomposite films were found to be suitable for food packaging applications. In another study by the same group, cellulose nanofiber/silver nanoparticle nanocomposites were synthesized via a UV irradiation-mediated synthesis [121]. Like in the previous study, the coating of the cellulose nanofibrils with the silver nanoparticles diminished the hydrogen bonding of the cellulose. The composite films were found to be antimicrobial when tested against *Escherichia coli* and *Staphylococcus aureus*. The silver nanoparticles were found to be released from the nanoparticles, which then interacted with *Staphylococcus aureus*. It was interesting to note that at lower concentrations, the nanocomposites were non-toxic. However, at concentrations ≥500 μg/mL, the nanocomposites showed considerable cytotoxicity in the human colon cells. The authors proposed that the synthesized nanocomposites can be exploited as nanofillers for the development of food packaging systems.

Nanocomposite films of polydiacetylene/silver nanoparticle/carboxymethyl cellulose films were also described in [122]. The presence of the silver nanoparticles in the films was confirmed by the scanning electron micrographs, while the core-shell structure of polydiacetylene/silver nanoparticles within the nanocomposites was established by the transmission electron micrographs. These films could act as time-temperature indicators. The authors reported that the developed films could be explored for monitoring the quality of the food products like fruits and vegetables. The films were able to change their color due to the progress of time. The change in the color information can allow the consumers to judge the quality of the food products. The color change was explained by the higher thermal conductivity of the silver nanoparticles and exposure of the large surface of polydiacetylene to higher temperatures. The addition of glycerol as the plasticizer delayed the change in the color of the nanocomposite films. Essentially, the authors concluded that the polydiacetylene/silver nanoparticle/carboxymethyl cellulose films could be explored as intelligent food packaging systems.

### 5.2. Cellulose and Cellulose-Based Nanostructure Containing Food Packaging Systems

Nanocomposites containing cellulose in both the continuum and dispersed phase were developed in [123]. The reinforcing phase was composed of cellulose nanocrystal, while the continuum phase was composed of 2,2,6,6-tetramethylpiperidine-1-oxy-oxidized cellulose nanofibers (TEMPO-CNFs). The nanocomposite film matrix was prepared by the simple vacuum filtration method. The films were transparent and free-standing matrixes. The films’ mechanical and water vapor barrier properties were improved by coating the nanocomposite films with cellulose nanocrystals on both sides. The developed films were resistant to several solvents (e.g., water, ethanol, tetrahydrofuran, and acetone) and were proposed for serval applications, including food packaging. In another experiment, thin nanocomposite films of cellulose nanofibers and cellulose nanocrystals were prepared that have the potential to be used for food packaging applications [124]. An increase in the cellulose nanocrystal content in the films improved the mechanical and optical properties of the films. Further, the crystallinity of the films was also increased. On the other hand, an increase in the cellulose nanofiber content improved the thermal stability of the nanocomposite films. The prepared films showed uniform mechanical properties in all directions.

Sá et al. (2020) reported the extraction and/or synthesis of lignin and cellulose nanocrystals from the cashew tree pruning fiber [125]. Further, bacterial cellulose was also extracted from the cashew apple juice. Thereafter, bacterial cellulose was then used to develop films. The films were then reinforced either by lignin, cellulose nanocrystals, or their combinations. The lignin and bacterial cellulose + lignin-containing films were brown, while the film that only contained bacterial cellulose had a whitish tinge. The presence of lignin in the bacterial cellulose film provided UV-protecting and antioxidant properties, which may make the films suitable for food packaging applications. It is interesting to note that none of the films were found to possess anti-microbial properties.

In [126], cellulose nanocrystals were used to reinforce the carboxymethyl cellulose/agar blend films. The cellulose nanocrystals were synthesized from onion peel. The use of the cellulose nanocrystals improved the physical properties of the films, including an improvement in the water resistance and mechanical properties. Also, the films were loaded with shikonin, which was isolated from the roots of *L. erythrorhizon*. The addition of this natural excipient allowed pH-responsive color change of the films. Further, this excipient imparted antioxidant and antibacterial properties to the nanocomposite films. It was proposed that the proposed active food packaging system could be used for analyzing the quality of food products.

All-cellulose nanocomposite films were proposed for food packaging applications in [127]. The films were made of cellulose nanofibers that were synthesized from bagasse. A suspension of the nanofibers was converted into nanofiber sheets by the vacuuming method. This was followed by drying under hot press conditions, wherein a 2 MPa pressure was applied for 1 h at a temperature of 100 °C. The thickness of the resultant nanofiber sheets varied in the range of 50–70 μm. Consequently, the formed nanofibrous sheets were used for the synthesis of the nanocomposites. At the initial stage, the sheets were activated using distilled water, acetone, and dimethylacetamide, followed by the treatment using a solution of dimethylacetamide and lithium chloride and subsequent washing. Finally, the nanofibrous sheets were then dried under hot press under 2 MPa pressure and 78 °C temperature for a period of 1 h to develop the all-cellulose nanocomposites. The prepared nanocomposite films showed good water vapor permeability. Further, the mechanical property of the nanocomposite films was found to be lower as compared to pure cellulose nanofiber sheets. This was related to the apparent crystallinity and crystallite size. The authors concluded that the all-cellulose nanocomposite films had sufficient properties to be explored as multi-performance material in various applications including food packaging. In [128], biodegradable nanocomposite films of gluten/ carboxymethyl cellulose/ cellulose nanofiber were prepared. The optimization of the films was achieved by the response surface methodology. During the optimization process, water vapor permeability as well asmechanical and color properties were considered. An increase in the cellulose nanofiber content increased the surface roughness of the developed nanocomposite films, which was associated with the formation of aggregates and holes when cellulose nanofibers were incorporated within the polymer blend matrix at high concentration. The addition of the cellulose nanofibers at optimum levels could considerably improve the cohesion within the nanocomposites. However, the films were found to be predominantly amorphous. The addition of the cellulose nanofibers did not induce the formation of the crystalline regions within the cellulosic matrices. Zabihollahi et al. (2020) reported the synthesis of carboxymethyl cellulose-based nanocomposite films [129]. The films were incorporated with *Lactobacillus plantarum*, cellulose nanofiber, and inulin. It was observed that the compatibility among the carboxymethyl cellulose, cellulose nanofiber, and inulin was quite good. Due to this reason, the nanocomposite films exhibited improved barrier and mechanical properties. It was also reported that the addition of inulin significantly improved the viability of the *Lactobacillus plantarum* within the nanocomposite films. This suggested that inulin acted as a prebiotic during the storage period. The films exhibited excellent antibacterial activity against many pathogenic bacterias. Finally, it was observed that the films were able to enhance the shelf life of chicken fillets when they were wrapped with the developed nanocomposite films. This confirmed the ability of the developed film to be explored as active food packaging material.

### 5.3. Cellulose and Montmorillonite Nanoclay Containing Food Packaging Systems

Nanocomposites of montmorillonite and chitin/cellulose blends were proposed in [130] by the solution casting method. The films were explored for their potential as food packaging material. The chitin and cellulose macromolecules were miscible with each other. The XRD analysis suggested that the polymer blend was able to penetrate within the interlayers of montmorillonite nanoclay. The homogenous dispersion of montmorillonite allowed efficient stress transfer. In [131], nanocomposites of cellulose and octadecylamine-modified montmorillonite were developed for food packaging and biomedical applications. The prepared films were able to interrupt the quorum-sensing-regulated physiological process of bacteria. Depending on the amount of octadecylamine-modified montmorillonite within the polymer matrices, the modified nanoclay was either exfoliated or intercalated. The anti-quorum-sensing activity of the nanocomposite films was tested successfully against the bacteria *C. violaceum*. The nanocomposite with the least amount of the modified nanoclay exhibited significantly higher anti-quorum-sensing. This observation was explained by the exfoliation of modified nanoclay. Further, it was noticed that the vitality of the bacteria was not affected by the prepared nanocomposites.

In [132], carboxymethyl cellulose (a synthetically modified cellulose), montmorillonite, and ε-poly-(l-lysine)-based ternary nanocomposites were prepared by the solution casting method. The UV barrier property of the carboxymethyl cellulose film was improved upon the addition of montmorillonite, and ε-poly-(l-lysine). The developed nanocomposites also showed improved tensile strength, hydrophobicity (analyzed through water contact angle), and water vapor barrier properties over the pristine carboxymethyl cellulose film that was used as the control. The addition of ε-poly-(l-lysine) into the nanocomposite films imparted antimicrobial properties to the films. The films presented not only antimicrobial properties against Gram-positive (*Staphylococus aureus*) and Gram-negative (*Escherichia coli*) bacteria but also fungi (*Botrytis cinerea* and *Rhizopus oligosporus*). The prepared films could extend the shelf life of strawberries for a period of 2 days. Zahedi et al. (2018) previously reported nanocomposite films of carboxymethyl cellulose in a similar study. The nanocomposite films consisted of hybrid montmorillonite/zinc oxide as the reinforcing agent. The inclusion of the nanoparticles could significantly hamper the water vapor permeability of the films and improve the ultimate tensile strength and the UV-blocking properties of the films. All of these properties are important parameters for a material to be used as a food packaging material. The increase in the tensile strength of the nanocomposite films can be explained by the formation of hydrogen bonds among the hydroxyl groups of carboxymethyl cellulose and montmorillonite. The films showed good antimicrobial properties against Gram-positive *S. aureus*, and Gram-negative *E. Coli*, which was attributed to the presence of zinc oxide nanoparticles. The authors concluded that the nanocomposite films showed sufficient properties to be explored for food packaging applications. In another study, nanocomposite films of carboxymethyl cellulose reinforced with modified Cloisite 30B and montmorillonite clay nanoparticles were proposed for food packaging applications [133]. The modification of the nanoclays was carried out by the silver and copper ions. The ions were present interlayer space of the nanoclays, which ultimately increased the interlayer spacing. The surface morphology of the pristine carboxymethyl cellulose film was smooth and homogenous. However, the addition of the nanoclays increased the heterogeneity of the film matrices. The films reinforced with modified montmorillonite were more heterogeneous than the films containing modified Cloisite 30B. Also, the mechanical strength of the nanocomposite films containing modified Cloisite 30B was better than the modified montmorillonite containing nanocomposite films. The inclusion of the reinforcing agents improved the UV barrier properties and correspondingly decreased the water permeation properties of the films. The antimicrobial properties of films that consisted of silver-modified clays were better than the films that contained copper-modified clays. It was concluded that the developed nanocomposite films were suitable for food packaging applications. In [134], novel ternary phase nanocomposites were developed by the solution casting method. The polymer matrix of the nanocomposite was composed of carboxymethyl cellulose, while the reinforced phase was a combination of sodium montmorillonite and titanium dioxide nanoparticles. The addition of the nanoparticles lowered the water vapor permeability of the carboxymethyl cellulose films. On the contrary, moisture content, mechanical stability, and thermal stability were increased. The improvement in the properties of the nanocomposite films was reasoned to the creation of hydrogen bonds amongst carboxymethyl cellulose and the nanoparticles. Titanium dioxide nanoparticles significantly incremented the UV-light blocking property of the carboxymethyl cellulose films. The nanocomposite films were found to have excellent properties to be explored as biodegradable packaging material.

Recently, disintegrated bacterial cellulose films that were reinforced with montmorillonite were proposed for food packaging applications in [135]. The use of disintegrated bacterial cellulose improved the thermal stability and the water vapor permeability of the pristine bacterial cellulose film. Interestingly, the tensile strength of the film was also reduced considerably. When disintegrated bacterial cellulose was employed, the changes in the film were explained by the alteration in the hydrogen bond network. This, in turn, caused a partial transformation from I_α_ to I_β_ allomorph. The montmorillonite and glycerol (plasticizer) improved the barrier properties of the nanocomposite films. This was explained by the formation of hydrogen bonds amongst the different components of the nanocomposite films. The prepared films were reported to be suitable as food packaging material.

A summary of the cellulose-based nanocomposites is compiled in Table 3.

Although we have discussed the use of polysaccharides for developing various nanocomposite materials in the aforesaid sections, it was not possible to discuss a wide variety of other polysaccharide-based nanocomposites as materials for food packaging applications. Accordingly, in Table 4, an overview of different studies on polysaccharide-based nanocomposite materials for food packaging applications is presented.

## 6. Conclusions

The materials for the packaging of the food play an important role not only in food security but also in food safety. Nowadays, most conventional food packaging systems are developed using synthetic polymers produced from non-renewable raw materials. Many researchers have proposed the use of polymers of natural origin. Among the various polymer categories, polysaccharides that are widely available in nature have gained much importance. However, pristine polysaccharide films have several disadvantages. Accordingly, polysaccharides were blended with several other polymers or reinforced with nanomaterials to improve the physical properties of the polysaccharide films. The inclusion of nanomaterials within the polysaccharide matrix or matrices of polysaccharide blends is regarded as a nanocomposite. Since these nanocomposites are biodegradable, they are also regarded as bio-nanocomposites. Such composite materials can be explored as an eco-friendly alternative to conventional synthetic polymers. Additionally, the nanocomposites of polysaccharides are an economically available and desirable “green packing product.” A scientometric analysis of the research articles suggested that the most commonly used polysaccharides for developing nanocomposites were chitosan, starch, and cellulose. Similarly, silver nanoparticles, cellulose nanostructures, and montmorillonite nanoclay were the preferred reinforcing agents to develop polysaccharide-based nanocomposites. The silver nanoparticle-containing nanocomposites exhibited superior antimicrobial properties. The addition of the reinforcing agents reduces the water permeability of the polysaccharide films and a consequent increment in moisture resistance. It was also observed that the nanocomposites’ thermal and mechanical properties were far superior to the pristine polysaccharide films. The improvement in the properties of the films is mainly reasoned either by the hydrogen bonding or physical intercalation among the components of the nanocomposite films. It can be summarized that polysaccharide-based nanocomposites have shown great promise as materials for food packaging applications.

The search for active, intelligent, and functional packaging solutions meets the unflagging demand of the packaging industry. Renewable, sustainable solutions, but above all, safe in direct contact with the product, are constantly sought after. Continuous data analysis on this topic provides scientific knowledge and information about potential development directions for the industry. When using new packaging materials for contact with food, two critical issues should be considered. Firstly, whether the packaging material was made of raw materials that are included in the positive list of approved materials; secondly, whether the current legal requirements are detailed enough to test the packaging material. This type of problem may especially arise in the case of nanomaterials, for which general and specific legal requirements may not cover such circumstances.

## Figures and Tables

**Table 1:** Summary of chitosan-based nanocomposites.

	Polysaccharide	Reinforcing Agent	Antimicrobial Activity	Food Tested	Reference
1.	Chitosan lactate	Silver nanoparticle	*E. coli*	--	[38]
2.	Chitosan	Biogenic silver nanoparticle	*E. coli*, *P. aeruginosa*, *B. subtilis*, *S. aureus*	--	[41]
3.	Chitosan	Silver nanoparticle within graphene oxide	*E. coli and S. aureus*	--	[45]
4.	Chitosan/gelatin	Silver nanoparticle	*--*	Green grapes	[47]
5.	Chitosan	Silver nanoparticle	*E. coli*, *Salmonella*, *S. aureus*, *L. monocytogenes*	--	[48]
6.	Chitosan	Cellulose nanocrystal	*--*	--	[51]
7.	Chitosan/guar gum	Cellulose nanocrystal	*--*	--	[52]
8.	Chitosan	Cellulose nanocrystal	*--*	--	[53]
9.	Chitosan	Cellulose nanocrystal	*--*	--	[54]
10.	Chitosan	Cellulose nanocrystal	*--*	--	[55]
11.	Chitosan	Montmorillonite	*--*	--	[65]
12.	Chitosan	Montmorillonite	Coliforms	Fresh poultry meat	[66]
13.	Chitosan	Montmorillonite	*--*	--	[67]
14.	Chitosan	Acidified modified montmorillonite	*S. aureus*, *E. coli*	--	[68]
15.	Chitosan nanoparticles/ chitosan	Montmorillonite	*--*	Sliced dry-cured ham	[72]

**Table 2:** Summary of starch-based nanocomposites.

	Polysaccharide	Reinforcing Agent	Antimicrobial Activity	Food Tested	Reference
1.	Starch	Silver nanoparticle	*--*	--	[81]
2.	Hydrophobic Noncrystalline Porous Starch	Silver nanoparticle	*E. coli*, *S, aureus*	--	[82]
3.	Sugar palm starch	Silver nanoparticle	*E. coli*, *S, aureus*, *S. cholerasuis*	--	[83]
4.	Amazonian tuber starch	Silver nanoparticle	*E. coli*, *S, aureus*	--	[84]
5.	Starch	Silver nanoparticle	*E. coli*, *Salmonella spp.*	Fresh cheese	[85]
6.	Native rice starch	Nanocellulose extracted from banana pseudostems	*--*	--	[94]
7.	Thermoplastic starch	Cellulose nanofiber	*--*	--	[95]
8.	Starch	Silver nanoparticles immobilized on cellulose nanofibrils	*E. coli*, *S, aureus*	--	[96]
9.	Starch	Carrot cellulose nano fibers	*E. coli*, *L. monocytogenes*, *S. typhimurium*, *Penicillium spp*.	Grapes	[97]
10.	Plasticised sugar palm starch	Nanofibrillated celluloses from sugar palm fibres	*--*	--	[98]
11.	Iranian potato starch	Montmorillonite	*--*	--	[104]
12.	Cationic starch	Nanoclay montmorillonite and ZnO nanopowders	*--*	--	[105]
13.	Starch	Montmorillonite	*E. coli*	--	[106]
14.	Thermoplastic starch	Montmorillonite	*Botrytis cinerea*	Strawberries	[107]
15.	Cassava starch and polycaprolactone	Sodium montmorillonite	*--*	--	[108]

**Table 3:** Summary of cellulose-based nanocomposites.

	Polysaccharide	Reinforcing Agent	Antimicrobial Activity	Food Tested	Reference
1.	Cellulose	Silver nanoparticle	*S. aureus*, *E. coli*	--	[116]
2.	Bacterial cellulose	Silver nanoparticle	*--*	--	[117]
3.	Regenerated cellulose	Amino-terminated hyperbranched polyamic anchored nanosilver	*S. aureus*, *E. coli*	--	[118]
4.	Cellulose acetate	Silver nanoparticle	*S. aureus*, *B. cereus*, *S. typhi*, *E. coli*, *K. pneumoniae*, *Pseudomonase spp*.	--	[119]
5.	Cellulose nanofibrils	Silver nanoparticle	*E. coli*, *L. monocytogenes*	--	[120]
6.	Cellulose nanofibers	Cellulose nanocrystals	*--*	--	[124]
7.	Bacterial cellulose	Lignin and cellulose nanocrystals from cashew tree pruning fiber	*S. aureus*, *E. Coli*, *S. cerevisiae*	--	[125]
8.	Carboxymethyl cellulose/agar	Cellulose nanocrystals	*--*	--	[126]
9.	Sugarcane bagasse nanofibers	*--*	--	--	[127]
10.	Gluten/ carboxymethyl cellulose	Cellulose nanofiber	*--*	--	[128]
11.	Chitin/cellulose	Montmorillonite nanoclay	*--*	--	[130]
12.	Cellulose	Octadecylamine-modified montmorillonite	*C. violaceum*	--	[131]
13.	Carboxymethyl cellulose	Montmorillonite and ε-poly-l-lysine	*S. aureus*, *E. Coli*, *B. Cinerea*, *R. oligosporus*	Strawberries	[132]
14.	Carboxymethyl cellulose	Cloisite 30B, montmorillonite	*S. aureus*, *E. Coli*	--	[133]
15.	Carboxymethyl cellulose	sodium montmorillonite, titanium dioxide	*--*	--	[134]

**Table 4:** Selected studies on polysaccharide-based nanocomposite materials with reinforcing agent, and antimicrobial activity for food packaging applications.

	Polysaccharide	Reinforcing Agent	Key Properties	Antimicrobial Activity	Food Tested	Reference
1.	Soluble soybean polysaccharide	Titanium dioxide	Antimicrobial activity; neither anti-cancerous nor pro-cancerous	*S. aureus* PTCC 1431	--	[136]
2.	Soluble soybean polysaccharide	Nano zinc oxide, microfibrillated cellulose	Antimicrobial activity	*E. coli*, *B. subtlis*	--	[137]
3.	Chitosan/agarose	Silver	Antibacterial activity	*P. aeruginosa*, *E. coli*, *S. aureus*	--	[138]
4.	Jackfruit filum polysaccharide	Titanium dioxide	Biodegradable; decreased the transparency, moisture content, and total soluble matter	*E. coli*, *S. aureus*	--	[139]
5.	Soluble soy bean polysaccharide	Cloisite 30B	Antibacterial activity; only packaging of solids foods such as bread	*S. typhi*, *S. epidermis*, *L. monocytogenes*, *A. niger*	Bread	[140]
6.	Soluble soy bean polysaccharide	Silica nanoparticles	Antibacterial activity; water solubility, and water vapor permeability decreased; mechanical performance increased	*S. aureus*, *B. subtilis*, *S. epidermidis*	Shrimp	[141]
7.	Protein/polysaccharide from the marine red alga (*Gracilaria lemaneiformis*)	Titanium dioxide	Antibacterial activity; nutrition retention and quality improvement of food products	*E. coli*, *S. aureus*	Cherry tomatoes	[142]
8.	Cationic guar gum	TEMPO-oxidized cellulose nanofibers	Maintained mooncake’s freshness	*--*	Mooncakes	[143]
9.	Soluble soybean polysaccharide	Montmorillonite	Antimicrobial activity against bacteria and mold; storage modulus of nanocomposites increased	*B. cereus*, *S. aureus*, *E. coli*, *P. expansum*	--	[144]
10.	Gum arabic, gum karaya, Kondagogu gum	Graphene oxide	Oxygen gas barriers; freestanding film with substantial mechanical strength	*--*	--	[145]
11.	Soluble soybean polysaccharide	Titanium dioxide	Antimicrobial activity; decreased water solubility, moisture content, and water-vapor permeability	*S. epidermidis*, *S. epidermis*, *L. monocytogenes*, *P. expansum*	--	[146]
12.	Curdlan	Glycyrrhiza polysaccharides-stabilized silver nanoparticles	Antimicrobial activity	*E. coli*	--	[147]
13.	Thermoplastic starch	Waxy starch nanocrystals	Effective interfacial hydrogen bonding between the polymer and the reinforcing agent	*--*	--	[148]
14.	Alginate, chitosan	Cellulose nanofiber	Higher tensile strength and better water barrier property	*--*	--	[149]
15.	Konjac glucomannan/gelatin	Rutin functionalized cellulose nanocrystal	Antibacterial activity; higher mechanical and barrier properties	*S. aureus*, *E. coli*	--	[150]
16.	Starch/kefiran	Zinc oxide nanoparticles	Antibacterial activity; higher mechanical properties; decreased water-related barrier properties	*--*	--	[151]
17.	Agar/carboxymethyl cellulose	Silver modified montmorillonite	Antibacterial activity	*B. subtilis*, *E. coli*	--	[152]
18.	Starch/kefiran	Titanium dioxide	Higher tensile strength; decreased water-related barrier properties	*--*	--	[153]
19.	Konjac glucomannan	Graphite carbon nitride nanosheets/ molybdenum disulfide nanodots	Sustained and efficient antimicrobial activity; Hemo- and cytocompatible	*S. aureus*, *E. coli*	Cherry tomatoes	[154]
20.	Cellulose acetate	Silver nanoparticles /gelatin-modified montmorillonite nanofiller	Antimicrobial and antioxidant properties	*E. coli*	--	[155]
21.	Agar/sodium alginate	Nano-silica	Enhanced mechanical properties, water resistance, and thermal stability	*--*	--	[156]
22.	Konjac glucomannan	Chitosan/gallic acid nanoparticles	Enhanced mechanical and barrier properties; Excellent mechanical properties	*S. aureus*, *E. coli*	--	[157]
23.	Soluble soybean polysaccharide	Silver nanoparticles	Excellent antibacterial property	*E. coli*, *S. aureus*, *P. aeruginosa*	--	[158]
24.	*Salvia macrosiphon* seed mucilage	Nanoclay	Increased hydrophobicity; antimicrobial property	*S. aureus*, *E. coli*	--	[159]
25.	Sweet potato starch/ lemon-waste pectin	Nano-titania	Biodegradable packaging material; increased thermal stability	*--*	--	[160]

## References

[1] Dash K.K., Ali N.A., Das D., Mohanta D. (2019). Thorough evaluation of sweet potato starch and lemon-waste pectin based-edible films with nano-titania inclusions for food packaging applications. Int. J. Biol. Macromol..

[2] Czarnecka-Komorowska D., Wiszumirska K. (2020). Sustainability design of plastic packaging for the Circular Economy. Polimery.

[3] Assman K., Klepka T. (2017). Biodegradable polymers—Application examples. Modern Polymer Materials and Their Processing.

[4] Wojciechowska P., Kozak W. (2016). Investigation of Transmission Oxygen Rate of Hybrid Materials Using Optical Method. Pol. J. Commod. Sci..

[5] Kozak W. (2020). Oxygen measurements in quality and safety assessment of products and services. Pol. J. Commod. Sci..

[6] Singh A., Shi Y., Magreault P., Kitts D.D., Jarzębski M., Siejak P., Pratap-Singh A. (2021). A Rapid Gas-Chromatography/Mass-Spectrometry Technique for Determining Odour Activity Values of Volatile Compounds in Plant Proteins: Soy, and Allergen-Free Pea and Brown Rice Protein. Molecules.

[7] Jamróz E., Kopel P. (2020). Polysaccharide and Protein Films with Antimicrobial/Antioxidant Activity in the Food Industry: A Review. Polymers.

[8] Guo Q., Liu Y., Cui S.W. (2021). Structure, classification and modification of polysaccharides. Comprehensive Glycoscience.

[9] Liu X., Xu Y., Zhan X., Xie W., Yang X., Cui S.W., Xia W. (2020). Development and properties of new kojic acid and chitosan composite biodegradable films for active packaging materials. Int. J. Biol. Macromol..

[10] Sothornvit R., Krochta J.M. (2005). Plasticizers in edible films and coatings. Innov. Food Packag..

[11] Rodríguez-Núñez J.R., Madera-Santana T.J., Sánchez-Machado D.I., López-Cervantes J., Valdez H.S. (2014). Chitosan/Hydrophilic Plasticizer-Based Films: Preparation, Physicochemical and Antimicrobial Properties. J. Polym. Environ..

[12] Chan-Matú D.I., Toledo-López V.M., de Vargas M.L.V.Y., Rincón-Arriaga S., Rodríguez-Félix A., Madera-Santana T.J. (2021). Preparation and characterization of chitosan-based bioactive films incorporating Moringa oleifera leaves extract. J. Food Meas. Charact..

[13] Zafar R., Zia K.M., Tabasum S., Jabeen F., Noreen A., Zuber M. (2016). Polysaccharide based bionanocomposites, properties and applications: A review. Int. J. Biol. Macromol..

[14] Xing W., Tang Y. (2021). On mechanical properties of nanocomposite hydrogels: Searching for superior properties. Nano Mater. Sci..

[15] Hallad S.A., Banapurmath N.R., Khan T.M.Y., Umarfarooq M.A., Soudagar M.E.M., Hunashyal A.M., Gujjar S.V., Yaradoddi J.S., Ganachari S.V., Elfasakhany A. (2021). Statistical Analysis of Polymer Nanocomposites for Mechanical Properties. Molecules.

[16] Lichade K.M., Jiang Y., Pan Y. (2021). Hierarchical Nano/Micro-Structured Surfaces With High Surface Area/Volume Ratios. J. Manuf. Sci. Eng..

[17] Brandelli A., Lopes N.A. (2021). Nanocomposite antimicrobial films based on biopolymers. Biopolymer-Based Nano Films.

[18] Sharma R., Jafari S.M., Sharma S. (2020). Antimicrobial bio-nanocomposites and their potential applications in food packaging. Food Control.

[19] Xia G.-X., Wu Y.-M., Bi Y.-F., Chen K., Zhang W.-W., Liu S.-Q., Zhang W.-J., Liu R.-H. (2021). Antimicrobial Properties and Application of Polysaccharides and Their Derivatives. Chin. J. Polym. Sci..

[20] Zheng Y., Monty J., Linhardt R.J. (2015). Polysaccharide-based nanocomposites and their applications. Carbohydr. Res..

[21] Xiao L. (2018). Chitosan Application in Textile Processing. Curr. Trends Fash. Technol. Text. Eng..

[22] Malerba M., Cerana R. (2018). Recent Advances of Chitosan Applications in Plants. Polymers.

[23] Kabanov V.L., Novinyuk L.V. (2020). Chitosan application in food technology: A review of rescent advances. Food Syst..

[24] Kumar M.N.R. (2000). A review of chitin and chitosan applications. React. Funct. Polym..

[25] Irastorza A., Zarandona I., Andonegi M., Guerrero P., de la Caba K. (2021). The versatility of collagen and chitosan: From food to biomedical applications. Food Hydrocoll..

[26] Schmitz C., Auza L.G., Koberidze D., Rasche S., Fischer R., Bortesi L. (2019). Conversion of Chitin to Defined Chitosan Oligomers: Current Status and Future Prospects. Mar. Drugs.

[27] Liu Z., Sun X. (2020). A Critical Review of the Abilities, Determinants, and Possible Molecular Mechanisms of Seaweed Polysaccharides Antioxidants. Int. J. Mol. Sci..

[28] Elfirta R.R., Saskiawan I. (2020). The functional character of Auricularia auricula crude polysaccharides: Antioxidant and antibacterial activity. Ber. Biol..

[29] Mo X., Liu Y., Li T., Peng W., Hu M., Wu C. (2017). Extraction optimization and characterization of polysaccharide antioxidants from *Pinellia ternata* (Thunb) Breit rhizome. Trop. J. Pharm. Res..

[30] Mu S., Yang W., Huang G. (2021). Antioxidant activities and mechanisms of polysaccharides. Chem. Biol. Drug Des..

[31] Raafat D., Sahl H.-G. (2009). Chitosan and its antimicrobial potential—A critical literature survey. Microb. Biotechnol..

[32] Kittur F.S., Kumar K.R., Tharanathan R.N. (1998). Functional packaging properties of chitosan films. Z. Lebensm. Forsch. A.

[33] Kurek M., Ščetar M., Voilley A., Galić K., Debeaufort F. (2012). Barrier properties of chitosan coated polyethylene. J. Membr. Sci..

[34] Darie-Niță R.N., Râpă M., Sivertsvik M., Rosnes J.T., Popa E.E., Dumitriu R.P., Marincaș O., Matei E., Predescu C., Vasile C. (2021). PLA-Based Materials Containing Bio-Plasticizers and Chitosan Modified with Rosehip Seed Oil for Ecological Packaging. Polymers.

[35] Kumar S., Mukherjee A., Dutta J. (2020). Chitosan based nanocomposite films and coatings: Emerging antimicrobial food packaging alternatives. Trends Food Sci. Technol..

[36] Fernandez J.G., Ingber D.E. (2011). Unexpected Strength and Toughness in Chitosan-Fibroin Laminates Inspired by Insect Cuticle. Adv. Mater..

[37] Da Silva Alves D.C., Healy B., de Pinto L.A.A., Cadaval T.R.S., Breslin C.B. (2021). Recent Developments in Chitosan-Based Adsorbents for the Removal of Pollutants from Aqueous Environments. Molecules.

[38] Ke C.-L., Deng F.-S., Chuang C.-Y., Lin C.-H. (2021). Antimicrobial Actions and Applications of Chitosan. Polymers.

[39] Tankhiwale R., Bajpai S.K. (2010). Silver-nanoparticle-loaded chitosan lactate films with fair antibacterial properties. J. Appl. Polym. Sci..

[40] Reidy B., Haase A., Luch A., Dawson K., Lynch I. (2013). Mechanisms of Silver Nanoparticle Release, Transformation and Toxicity: A Critical Review of Current Knowledge and Recommendations for Future Studies and Applications. Materials.

[41] Ahmad S.A., Das S.S., Khatoon A., Ansari M.T., Afzal M., Hasnain M.S., Nayak A.K. (2020). Bactericidal activity of silver nanoparticles: A mechanistic review. Mater. Sci. Energy Technol..

[42] Kadam D., Momin B., Palamthodi S., Lele S.S. (2019). Physicochemical and functional properties of chitosan-based nano-composite films incorporated with biogenic silver nanoparticles. Carbohydr. Polym..

[43] Lee S.H., Jun B.-H. (2019). Silver Nanoparticles: Synthesis and Application for Nanomedicine. Int. J. Mol. Sci..

[44] Anis A., Pal K., Al-Zahrani S.A. (2021). Essential Oil-Containing Polysaccharide-Based Edible Films and Coatings for Food Security Applications. Polymers.

[45] Wu Z., Huang X., Li Y.-C., Xiao H., Wang X. (2018). Novel chitosan films with laponite immobilized Ag nanoparticles for active food packaging. Carbohydr. Polym..

[46] Gu B., Jiang Q., Luo B., Liu C., Ren J., Wang X., Wang X. (2021). A sandwich-like chitosan-based antibacterial nanocomposite film with reduced graphene oxide immobilized silver nanoparticles. Carbohydr. Polym..

[47] Salleh A., Naomi R., Utami N.D., Mohammad A.W., Mahmoudi E., Mustafa N., Fauzi M.B. (2020). The Potential of Silver Nanoparticles for Antiviral and Antibacterial Applications: A Mechanism of Action. Nanomaterials.

[48] Kumar S., Shukla A., Baul P.P., Mitra A., Halder D. (2018). Biodegradable hybrid nanocomposites of chitosan/gelatin and silver nanoparticles for active food packaging applications. Food Packag. Shelf Life.

[49] Qin Y., Liu Y., Yuan L., Yong H., Liu J. (2019). Preparation and characterization of antioxidant, antimicrobial and pH-sensitive films based on chitosan, silver nanoparticles and purple corn extract. Food Hydrocoll..

[50] Ossai C.I., Raghavan N. (2018). Nanostructure and nanomaterial characterization, growth mechanisms, and applications. Nanotechnol. Rev..

[51] Celebi H., Kurt A. (2015). Effects of processing on the properties of chitosan/cellulose nanocrystal films. Carbohydr. Polym..

[52] Yadav M., Behera K., Chang Y.-H., Chiu F.-C. (2020). Cellulose Nanocrystal Reinforced Chitosan Based UV Barrier Composite Films for Sustainable Packaging. Polymers.

[53] Tang Y., Zhang X., Zhao R., Guo D., Zhang J. (2018). Preparation and properties of chitosan/guar gum/nanocrystalline cellulose nanocomposite films. Carbohydr. Polym..

[54] Kusmono K., Wildan M.W., Lubis F.I. (2021). Fabrication and Characterization of Chitosan/Cellulose Nanocrystal/Glycerol Bio-Composite Films. Polymers.

[55] De Andrade M.R., Nery T.B.R., de Santana T.I.D., Leal I.L., Rodrigues L.A.P., de Oliveira Reis J.H.D.O., Druzian J.I., Machado B.A.S., Andrade D. (2019). Effect of Cellulose Nanocrystals from Different Lignocellulosic Residues to Chitosan/Glycerol Films. Polymers.

[56] Xu Y., Willis S., Jordan K., Sismour E. (2018). Chitosan nanocomposite films incorporating cellulose nanocrystals and grape pomace extracts. Packag. Technol. Sci..

[57] Gan P.G., Sam S.T., Abdullah M.F., Omar M.F., Tan W.K. (2021). Water resistance and biodegradation properties of conventionally-heated and microwave-cured cross-linked cellulose nanocrystal/chitosan composite films. Polym. Degrad. Stab..

[58] Rojas J., Bedoya M., Ciro Y. (2015). Current trends in the production of cellulose nanoparticles and nanocomposites for biomedical applications. Cellulose—Fundamental Aspects and Current Trends.

[59] Salari M., Khiabani M.S., Mokarram R.R., Ghanbarzadeh B., Kafil H.S. (2018). Development and evaluation of chitosan based active nanocomposite films containing bacterial cellulose nanocrystals and silver nanoparticles. Food Hydrocoll..

[60] Thou C.Z., Khan F.S.A., Mubarak N.M., Ahmad A., Khalid M., Jagadish P., Walvekar R., Abdullah E.C., Khan S., Khan M. (2021). Surface charge on chitosan/cellulose nanowhiskers composite via functionalized and untreated carbon nanotube. Arab. J. Chem..

[61] Deng Z., Jung J., Simonsen J., Zhao Y. (2018). Cellulose nanocrystals Pickering emulsion incorporated chitosan coatings for improving storability of postharvest Bartlett pears (*Pyrus communis*) during long-term cold storage. Food Hydrocoll..

[62] Adel A.M., Ibrahim A.A., El-Shafei A.M., Al-Shemy M.T. (2019). Inclusion complex of clove oil with chitosan/β-cyclodextrin citrate/oxidized nanocellulose biocomposite for active food packaging. Food Packag. Shelf Life.

[63] Mohammadi Sadati S.M., Shahgholian-Ghahfarrokhi N., Shahrousvand E., Mohammadi-Rovshandeh J., Shahrousvand M. (2021). Edible chitosan/cellulose nanofiber nanocomposite films for potential use as food packaging. Mater. Technol..

[64] Souza V.G.L., Pires J.R.A., Rodrigues P.F., Lopes A.A.S., Fernandes F.M.B., Duarte M.P., Coelhoso I.M., Fernando A.L. (2018). Bionanocomposites of chitosan/montmorillonite incorporated with Rosmarinus officinalis essential oil: Development and physical characterization. Food Packag. Shelf Life.

[65] Pires J., Paula C.D., de Souza V.G.L., Fernando A.L., Coelhoso I. (2021). Understanding the Barrier and Mechanical Behavior of Different Nanofillers in Chitosan Films for Food Packaging. Polymers.

[66] Wang K., Zhao L., He B. (2021). Chitosan/Montmorillonite Coatings for the Fabrication of Food-Safe Greaseproof Paper. Polymers.

[67] Pires J.R.A., de Souza V.G.L., Fernando A.L. (2018). Chitosan/montmorillonite bionanocomposites incorporated with rosemary and ginger essential oil as packaging for fresh poultry meat. Food Packag. Shelf Life.

[68] Souza V.G.L., Pires J.R.A., Rodrigues C., Rodrigues P.F., Lopes A., Silva R.J., Caldeira J., Duarte M.P., Fernandes F.B., Coelhoso I.M. (2019). Physical and Morphological Characterization of Chitosan/Montmorillonite Films Incorporated with Ginger Essential Oil. Coatings.

[69] Cui R., Yan J., Cao J., Qin Y., Yuan M., Li L. (2021). Release properties of cinnamaldehyde loaded by montmorillonite in chitosan-based antibacterial food packaging. Int. J. Food Sci. Technol..

[70] Romero-Bastida C.A., Velazquez G., Bautista-Baños S. (2020). Effect of the preparation method on the properties of nanocomposites based on chitosan, montmorillonite and essential oils. Rev. Mex. Ing. Quím..

[71] Müller K., Bugnicourt E., Latorre M., Jorda M., Echegoyen Sanz Y., Lagaron J., Miesbauer O., Bianchin A., Hankin S., Bölz U. (2017). Review on the Processing and Properties of Polymer Nanocomposites and Nanocoatings and Their Applications in the Packaging, Automotive and Solar Energy Fields. Nanomaterials.

[72] Rodrigues C., de Mello J.M.M., Dalcanton F., Macuvele D.L.P., Padoin N., Fiori M.A., Soares C., Riella H.G. (2020). Mechanical, Thermal and Antimicrobial Properties of Chitosan-Based-Nanocomposite with Potential Applications for Food Packaging. J. Polym. Environ..

[73] Yan W., Chen W., Muhammad U., Zhang J., Zhuang H., Zhou G. (2019). Preparation of α-tocopherol-chitosan nanoparticles/chitosan/montmorillonite film and the antioxidant efficiency on sliced dry-cured ham. Food Control.

[74] Zhang L., Wang H., Jin C., Zhang R., Li L., Li X., Jiang S. (2017). Sodium lactate loaded chitosan-polyvinyl alcohol/montmorillonite composite film towards active food packaging. Innov. Food Sci. Emerg. Technol..

[75] Vianna T.C., Marinho C.O., Júnior L.M., Ibrahim S.A., Vieira R.P. (2021). Essential oils as additives in active starch-based food packaging films: A review. Int. J. Biol. Macromol..

[76] Cheng H., Chen L., McClements D.J., Yang T., Zhang Z., Ren F., Miao M., Tian Y., Jin Z. (2021). Starch-based biodegradable packaging materials: A review of their preparation, characterization and diverse applications in the food industry. Trends Food Sci. Technol..

[77] Mohammad F., Arfin T., Bwatanglang I.B., Al-lohedan H.A. (2019). Starch-based nanocomposites: Types and industrial applications. Bio-Based Polymers and Nanocomposites.

[78] Othman S.H., Othman N.F.L., Shapi’i R.A., Ariffin S.H., Yunos K.F.M. (2021). Corn Starch/Chitosan Nanoparticles/Thymol Bio-Nanocomposite Films for Potential Food Packaging Applications. Polymers.

[79] Yin Z., Zeng J., Wang C., Pan Z. (2015). Preparation and Properties of Cross-Linked Starch Nanocrystals/Polylactic Acid Nanocomposites. Int. J. Polym. Sci..

[80] Shah N., Mewada R.K., Mehta T. (2016). Crosslinking of starch and its effect on viscosity behaviour. Rev. Chem. Eng..

[81] Żołek-Tryznowska Z., Kałuża A. (2021). The Influence of Starch Origin on the Properties of Starch Films: Packaging Performance. Materials.

[82] Jung J., Raghavendra G.M., Kim D., Seo J. (2018). One-step synthesis of starch-silver nanoparticle solution and its application to antibacterial paper coating. Int. J. Biol. Macromol..

[83] Dang C., Yin Y., Xu M., Pu J. (2017). Hydrophobic Noncrystalline Porous Starch (NCPS): Dispersed Silver Nanoparticle Suspension as an Antibacterial Coating for Packaging Paper. BioResources.

[84] Rozilah A., Jaafar C.N.A., Sapuan S.M., Zainol I., Ilyas R.A. (2020). The Effects of Silver Nanoparticles Compositions on the Mechanical, Physiochemical, Antibacterial, and Morphology Properties of Sugar Palm Starch Biocomposites for Antibacterial Coating. Polymers.

[85] Silva L.S.C., Martim S.R., Gomes D.M.D., Prado F.B., Marinho N.M.V., de Silva T.A., Castillo T.A., de Rego J.A.R., do Seabra A.B., Durán N. (2021). Amazonian tuber starch based films incorporated with silver nanoparticles for preservation of fruits. Res. Soc. Dev..

[86] Ortega F., Giannuzzi L., Arce V.B., García M.A. (2017). Active composite starch films containing green synthetized silver nanoparticles. Food Hydrocoll..

[87] Mohseni M.S., Khalilzadeh M.A., Mohseni M., Hargalani F.Z., Getso M.I., Raissi V., Raiesi O. (2020). Green synthesis of Ag nanoparticles from pomegranate seeds extract and synthesis of Ag-Starch nanocomposite and characterization of mechanical properties of the films. Biocatal. Agric. Biotechnol..

[88] Ceballos R.L., von Bilderling C., Guz L., Bernal C., Famá L. (2021). Effect of greenly synthetized silver nanoparticles on the properties of active starch films obtained by extrusion and compression molding. Carbohydr. Polym..

[89] Ortega F., Arce V.B., Garcia M.A. (2021). Nanocomposite starch-based films containing silver nanoparticles synthesized with lemon juice as reducing and stabilizing agent. Carbohydr. Polym..

[90] Kumar R., Ghoshal G., Goyal M. (2020). Development and characterization of corn starch based nanocomposite film with AgNPs and plant extract. Mater. Sci. Energy Technol..

[91] Peighambardoust S.J., Peighambardoust S.H., Pournasir N., Mohammadzadeh Pakdel P. (2019). Properties of active starch-based films incorporating a combination of Ag, ZnO and CuO nanoparticles for potential use in food packaging applications. Food Packag. Shelf Life.

[92] Mahuwala A.A., Hemant V., Meharwade S.D., Deb A., Chakravorty A., Grace A.N., Raghavan V. (2020). Synthesis and characterisation of starch/agar nanocomposite films for food packaging application. IET Nanobiotechnol..

[93] Mathew S., Jayakumar A., Kumar V.P., Mathew J., Radhakrishnan E.K. (2019). One-step synthesis of eco-friendly boiled rice starch blended polyvinyl alcohol bionanocomposite films decorated with in situ generated silver nanoparticles for food packaging purpose. Int. J. Biol. Macromol..

[94] Srikhao N., Kasemsiri P., Ounkaew A., Lorwanishpaisarn N., Okhawilai M., Pongsa U., Hiziroglu S., Chindaprasirt P. (2021). Bioactive Nanocomposite Film Based on Cassava Starch/Polyvinyl Alcohol Containing Green Synthesized Silver Nanoparticles. J. Polym. Environ..

[95] Jeevahan J., Chandrasekaran M. (2020). Influence of Nanocellulose Additive on the Film Properties of Native Rice Starch-based Edible Films for Food Packaging. Recent Pat. Nanotechnol..

[96] Fazeli M., Keley M., Biazar E. (2018). Preparation and characterization of starch-based composite films reinforced by cellulose nanofibers. Int. J. Biol. Macromol..

[97] Yuan T., Zeng J., Wang B., Cheng Z., Gao W., Xu J., Chen K. (2021). Silver nanoparticles immobilized on cellulose nanofibrils for starch-based nanocomposites with high antibacterial, biocompatible, and mechanical properties. Cellulose.

[98] Ghoshal G., Singh D. (2020). Synthesis and characterization of starch nanocellulosic films incorporated with Eucalyptus globulus leaf extract. Int. J. Food Microbiol..

[99] Ilyas R.A., Sapuan S.M., Ibrahim R., Abral H., Ishak M.R., Zainudin E.S., Atiqah A., Atikah M.S.N., Syafri E., Asrofi M. (2020). Thermal, Biodegradability and Water Barrier Properties of Bio-Nanocomposites Based on Plasticised Sugar Palm Starch and Nanofibrillated Celluloses from Sugar Palm Fibres. J. Biobased Mater. Bioenergy.

[100] De Teixeira E.M., Pasquini D., Curvelo A.A.S., Corradini E., Belgacem M.N., Dufresne A. (2009). Cassava bagasse cellulose nanofibrils reinforced thermoplastic cassava starch. Carbohydr. Polym..

[101] Kaushik A., Kaur R., Tripathi S.K., Dharamvir K., Kumar R., Saini G.S.S. (2011). Thermal Behaviour of Nanocomposites based on Glycerol Plasticized Thermoplastic Starch and Cellulose Nanocrystallites. AIP Conf. Proc..

[102] Ilyas R.A., Sapuan S.M., Ishak M.R., Zainudin E.S. (2018). Development and characterization of sugar palm nanocrystalline cellulose reinforced sugar palm starch bionanocomposites. Carbohydr. Polym..

[103] Bagde P., Nadanathangam V. (2019). Mechanical, antibacterial and biodegradable properties of starch film containing bacteriocin immobilized crystalline nanocellulose. Carbohydr. Polym..

[104] Fabra M.J., López-Rubio A., Ambrosio-Martín J., Lagaron J.M. (2016). Improving the barrier properties of thermoplastic corn starch-based films containing bacterial cellulose nanowhiskers by means of PHA electrospun coatings of interest in food packaging. Food Hydrocoll..

[105] Oleyaei S.A., Moayedi A.A., Ghanbarzadeh B. (2017). The Effect of Montmorillonite (MMT) on Structural, Thermal and Optical Properties of Iranian Potato Starch Based Nanobiocomposite Films. Innov. Food Technol..

[106] Vaezi K., Asadpour G., Sharifi H. (2019). Effect of ZnO nanoparticles on the mechanical, barrier and optical properties of thermoplastic cationic starch/montmorillonite biodegradable films. Int. J. Biol. Macromol..

[107] De Souza A.G., dos Santos N.M.A., da Silva Torin R.F., dos Santos Rosa D. (2020). Synergic antimicrobial properties of Carvacrol essential oil and montmorillonite in biodegradable starch films. Int. J. Biol. Macromol..

[108] Campos-Requena V.H., Rivas B.L., Pérez M.A., Figueroa C.R., Figueroa N.E., Sanfuentes E.A. (2017). Thermoplastic starch/clay nanocomposites loaded with essential oil constituents as packaging for strawberries—In vivo antimicrobial synergy over Botrytis cinerea. Postharvest Biol. Technol..

[109] Piñeros-Guerrero N., Marsiglia-Fuentes R., Ortega-Toro R. (2021). Improvement of the physicochemical properties of composite materials based on cassavastarch and polycaprolactone reinforced with sodium montmorillonite. Rev. Mex. Ing. Quím..

[110] Kumar M., Panjagari N.R., Kanade P.P., Singh A.K., Badola R., Ganguly S., Behare P.V., Sharma R., Alam T. (2018). Sodium caseinate-starch-modified montmorillonite based biodegradable film: Laboratory food extruder assisted exfoliation and characterization. Food Packag. Shelf Life.

[111] Yousefi A.R., Savadkoohi B., Zahedi Y., Hatami M., Ako K. (2019). Fabrication and characterization of hybrid sodium montmorillonite/TiO_2_ reinforced cross-linked wheat starch-based nanocomposites. Int. J. Biol. Macromol..

[112] Mathew S., Snigdha S., Mathew J., Radhakrishnan E.K. (2018). Poly(vinyl alcohol): Montmorillonite: Boiled rice water (starch) blend film reinforced with silver nanoparticles; characterization and antibacterial properties. Appl. Clay Sci..

[113] Tayeb A., Amini E., Ghasemi S., Tajvidi M. (2018). Cellulose Nanomaterials—Binding Properties and Applications: A Review. Molecules.

[114] Wahid F., Zhong C. (2021). Production and applications of bacterial cellulose. Biomass, Biofuels, Biochemicals.

[115] Feng Y.H., Li J.C., Lin Q., Pang S.J., Wang X.B., Wu Z.X. (2007). The Characterizations of Bacterial Cellulose and Dialdehyde Celluloses from Bacterial Cellulose. Key Eng. Mater..

[116] Betlej I., Zakaria S., Krajewski K.J., Boruszewski P. (2021). Bacterial Cellulose—Properties and Its Potential Application. Sains Malays..

[117] Chen Q.-Y., Xiao S.-L., Shi S.Q., Cai L.-P. (2020). A One-Pot Synthesis and Characterization of Antibacterial Silver Nanoparticle–Cellulose Film. Polymers.

[118] Adepu S., Khandelwal M. (2018). Broad-spectrum antimicrobial activity of bacterial cellulose silver nanocomposites with sustained release. J. Mater. Sci..

[119] Gu R., Yun H., Chen L., Wang Q., Huang X. (2020). Regenerated Cellulose Films with Amino-Terminated Hyperbranched Polyamic Anchored Nanosilver for Active Food Packaging. ACS Appl. Bio Mater..

[120] Marrez D.A., Abdelhamid A.E., Darwesh O.M. (2019). Eco-friendly cellulose acetate green synthesized silver nano-composite as antibacterial packaging system for food safety. Food Packag. Shelf Life.

[121] Yu Z., Wang W., Kong F., Lin M., Mustapha A. (2019). Cellulose nanofibril/silver nanoparticle composite as an active food packaging system and its toxicity to human colon cells. Int. J. Biol. Macromol..

[122] Yu Z., Wang W., Dhital R., Kong F., Lin M., Mustapha A. (2019). Antimicrobial effect and toxicity of cellulose nanofibril/silver nanoparticle nanocomposites prepared by an ultraviolet irradiation method. Colloids Surf. B Biointerfaces.

[123] Saenjaiban A., Singtisan T., Suppakul P., Jantanasakulwong K., Punyodom W., Rachtanapun P. (2020). Novel Color Change Film as a Time–Temperature Indicator Using Polydiacetylene/Silver Nanoparticles Embedded in Carboxymethyl Cellulose. Polymers.

[124] Kwon G., Lee K., Kim D., Jeon Y., Kim U.-J., You J. (2020). Cellulose nanocrystal-coated TEMPO-oxidized cellulose nanofiber films for high performance all-cellulose nanocomposites. J. Hazard. Mater..

[125] Sun X., Wu Q., Zhang X., Ren S., Lei T., Li W., Xu G., Zhang Q. (2018). Nanocellulose films with combined cellulose nanofibers and nanocrystals: Tailored thermal, optical and mechanical properties. Cellulose.

[126] Sá N.M.S.M., Mattos A.L.A., Silva L.M.A., Brito E.S., Rosa M.F., Azeredo H.M.C. (2020). From cashew byproducts to biodegradable active materials: Bacterial cellulose-lignin-cellulose nanocrystal nanocomposite films. Int. J. Biol. Macromol..

[127] Roy S., Kim H.-J., Rhim J.-W. (2021). Synthesis of Carboxymethyl Cellulose and Agar-Based Multifunctional Films Reinforced with Cellulose Nanocrystals and Shikonin. ACS Appl. Polym. Mater..

[128] Ghaderi M., Mousavi M., Yousefi H., Labbafi M. (2014). All-cellulose nanocomposite film made from bagasse cellulose nanofibers for food packaging application. Carbohydr. Polym..

[129] Bagheri V., Ghanbarzadeh B., Ayaseh A., Ostadrahimi A., Ehsani A., Alizadeh-Sani M., Adun P.A. (2019). The optimization of physico-mechanical properties of bionanocomposite films based on gluten/ carboxymethyl cellulose/ cellulose nanofiber using response surface methodology. Polym. Test..

[130] Zabihollahi N., Alizadeh A., Almasi H., Hanifian S., Hamishekar H. (2020). Development and characterization of carboxymethyl cellulose based probiotic nanocomposite film containing cellulose nanofiber and inulin for chicken fillet shelf life extension. Int. J. Biol. Macromol..

[131] Lao T.L., Pengson L.T., Placido J., Diaz L.J. (2019). Synthesis of Montmorillonite Nanoclay Reinforced Chitin-cellulose Nanocomposite Film. IOP Conf. Ser. Mater. Sci. Eng..

[132] Demircan D., Ilk S., Zhang B. (2017). Cellulose-Organic Montmorillonite Nanocomposites as Biomacromolecular Quorum-Sensing Inhibitor. Biomacromolecules.

[133] He Y., Fei X., Li H. (2020). Carboxymethyl cellulose-based nanocomposites reinforced with montmorillonite and ε-poly-l-lysine for antimicrobial active food packaging. J. Appl. Polym. Sci..

[134] Peighambardoust S.J., Zahed-Karkaj S., Peighambardoust S.H., Ebrahimi Y., Peressini D. (2020). Characterization of carboxymethyl cellulose-based active films incorporating non-modified and Ag or Cu-modified Cloisite 30B and montmorillonite nanoclays. Iran. Polym. J..

[135] Achachlouei B.F., Zahedi Y. (2018). Fabrication and characterization of CMC-based nanocomposites reinforced with sodium montmorillonite and TiO_2_ nanomaterials. Carbohydr. Polym..

[136] Sommer A., Staroszczyk H., Sinkiewicz I., Bruździak P. (2021). Preparation and Characterization of Films Based on Disintegrated Bacterial Cellulose and Montmorillonite. J. Polym. Environ..

[137] Salarbashi D., Tafaghodi M., Bazzaz B.S.F. (2018). Soluble soybean polysaccharide/TiO_2_ bionanocomposite film for food application. Carbohydr. Polym..

[138] Liu J., Liu C., Zheng X., Chen M., Tang K. (2020). Soluble soybean polysaccharide/nano zinc oxide antimicrobial nanocomposite films reinforced with microfibrillated cellulose. Int. J. Biol. Macromol..

[139] Ghasemzadeh H., Afraz S., Moradi M., Hassanpour S. (2021). Antimicrobial chitosan-agarose full polysaccharide silver nanocomposite films. Int. J. Biol. Macromol..

[140] Jin B., Li X., Zhou X., Xu X., Jian H., Li M., Guo K., Guan J., Yan S. (2017). Fabrication and characterization of nanocomposite film made from a jackfruit filum polysaccharide incorporating TiO_2_ nanoparticles by photocatalysis. RSC Adv..

[141] Salarbashi D., Tafaghodi M., Bazzaz B.S.F., Birjand S.M.A., Bazeli J. (2018). Characterization of a green nanocomposite prepared from soluble soy bean polysaccharide/Cloisite 30B and evaluation of its toxicity. Int. J. Biol. Macromol..

[142] Salarbashi D., Tafaghodi M., Bazzaz B.S.F., Mohammad Aboutorabzade S., Fathi M. (2021). pH-sensitive soluble soybean polysaccharide/SiO 2 incorporated with curcumin for intelligent packaging applications. Food Sci. Nutr..

[143] He Q., Huang Y., Lin B., Wang S. (2017). A nanocomposite film fabricated with simultaneously extracted protein-polysaccharide from a marine alga and TiO_2_ nanoparticles. J. Appl. Phycol..

[144] Dai L., Xi X., Li X., Li W., Du Y., Lv Y., Wang W., Ni Y. (2021). Self-assembled all-polysaccharide hydrogel film for versatile paper-based food packaging. Carbohydr. Polym..

[145] Salarbashi D., Noghabi M.S., Bazzaz B.S.F., Shahabi-Ghahfarrokhi I., Jafari B., Ahmadi R. (2017). Eco-friendly soluble soybean polysaccharide/nanoclay Na+ bionanocomposite: Properties and characterization. Carbohydr. Polym..

[146] Venkateshaiah A., Cheong J.Y., Habel C., Wacławek S., Lederer T., Černík M., Kim I.-D., Padil V.V.T., Agarwal S. (2020). Tree Gum-Graphene Oxide Nanocomposite Films as Gas Barriers. ACS Appl. Nano Mater..

[147] Salarbashi D., Tafaghodi M., Bazzaz B.S.F., Jafari B. (2018). Characterization of soluble soybean (SSPS) polysaccharide and development of eco-friendly SSPS/TiO2 nanoparticle bionanocomposites. Int. J. Biol. Macromol..

[148] Cai Z., Dai Q., Guo Y., Wei Y., Wu M., Zhang H. (2019). Glycyrrhiza polysaccharide-mediated synthesis of silver nanoparticles and their use for the preparation of nanocomposite curdlan antibacterial film. Int. J. Biol. Macromol..

[149] González K., Iturriaga L., González A., Eceiza A., Gabilondo N. (2020). Improving mechanical and barrier properties of thermoplastic starch and polysaccharide nanocrystals nanocomposites. Eur. Polym. J..

[150] Zhao K., Wang W., Teng A., Zhang K., Ma Y., Duan S., Li S., Guo Y. (2020). Using cellulose nanofibers to reinforce polysaccharide films: Blending vs layer-by-layer casting. Carbohydr. Polym..

[151] Wang L., Lin L., Guo Y., Long J., Mu R.-J., Pang J. (2020). Enhanced functional properties of nanocomposite film incorporated with EGCG-loaded dialdehyde glucomannan/gelatin matrix for food packaging. Food Hydrocoll..

[152] Shahabi-Ghahfarrokhi I., Babaei-Ghazvini A. (2019). Using photo-modification to compatibilize nano-ZnO in development of starch-kefiran-ZnO green nanocomposite as food packaging material. Int. J. Biol. Macromol..

[153] Makwana D., Castaño J., Somani R.S., Bajaj H.C. (2020). Characterization of Agar-CMC/Ag-MMT nanocomposite and evaluation of antibacterial and mechanical properties for packaging applications. Arab. J. Chem..

[154] Goudarzi V., Shahabi-Ghahfarrokhi I. (2018). Development of photo-modified starch/kefiran/TiO_2_ bio-nanocomposite as an environmentally-friendly food packaging material. Int. J. Biol. Macromol..

[155] Ni Y., Sun J., Wang J. (2021). Enhanced antimicrobial activity of konjac glucomannan nanocomposite films for food packaging. Carbohydr. Polym..

[156] Dairi N., Ferfera-Harrar H., Ramos M., Garrigós M.C. (2019). Cellulose acetate/AgNPs-organoclay and/or thymol nano-biocomposite films with combined antimicrobial/antioxidant properties for active food packaging use. Int. J. Biol. Macromol..

[157] Hou X., Xue Z., Xia Y., Qin Y., Zhang G., Liu H., Li K. (2019). Effect of SiO_2_ nanoparticle on the physical and chemical properties of eco-friendly agar/sodium alginate nanocomposite film. Int. J. Biol. Macromol..

[158] Wu C., Li Y., Du Y., Wang L., Tong C., Hu Y., Pang J., Yan Z. (2019). Preparation and characterization of konjac glucomannan-based bionanocomposite film for active food packaging. Food Hydrocoll..

[159] Ma Z., Liu J., Liu Y., Zheng X., Tang K. (2021). Green synthesis of silver nanoparticles using soluble soybean polysaccharide and their application in antibacterial coatings. Int. J. Biol. Macromol..

[160] Davachi S.M., Shekarabi A.S. (2018). Preparation and characterization of antibacterial, eco-friendly edible nanocomposite films containing Salvia macrosiphon and nanoclay. Int. J. Biol. Macromol..

